# Short-chain fatty acids: key antiviral mediators of gut microbiota

**DOI:** 10.3389/fimmu.2025.1614879

**Published:** 2025-07-25

**Authors:** Zhiqiang Xu, Tao Wang, Yanjin Wang, Yongfeng Li, Yuan Sun, Hua-Ji Qiu

**Affiliations:** State Key Laboratory for Animal Disease Control and Prevention, National African Swine Fever Para-Reference Laboratory, National High Containment Facilities for Animal Diseases Control and Prevention, Harbin Veterinary Research Institute, Chinese Academy of Agricultural Sciences, Harbin, China

**Keywords:** antiviral immunity, G-protein-coupled receptor, gut microbiota, histone acetyltransferase, histone deacetylase, short-chain fatty acid

## Abstract

The effects of gut microbiota on antiviral immunity have been well-documented in recent years, whereas a mechanistic understanding of microbiota-derived metabolite-related signaling pathways is still lacking. Short-chain fatty acids (SCFAs), key metabolites produced by gut bacterial microbiota *via* dietary fiber fermentation and amino acid metabolism, have been shown to facilitate host antiviral responses. In this review, we summarized the detailed mechanisms which could contribute to the regulation of antiviral immunity engaged and initiated by SCFAs, involving G-protein-coupled receptor (GPCR)-mediated, histone deacetylase (HDAC)-mediated, and metabolic pathways. We also discuss the implications of SCFAs for viral disease management and pandemic preparedness. This review provides novel insights into the antiviral activities of SCFAs and highlights the therapeutic potential of SCFA-producing bacteria.

## Graphical Abstract

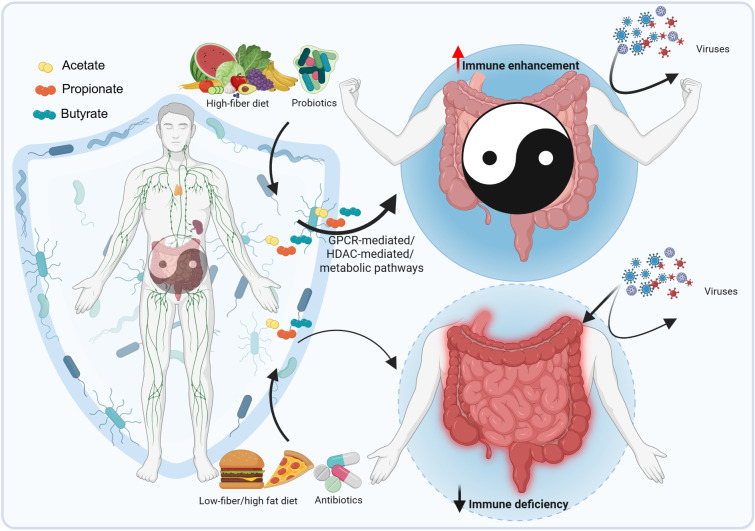

## 1 Introduction

The microbe communities in the gastrointestinal tract, which are collectively called “gut microbiota”, include bacteria, fungi, viruses, archaea, *etc.*, and bacteria are the largest component. In contrast, the “gut microbiome” encompasses the gut microbiota and their genomes (1, 2). The gut microbiota, especially short-chain fatty acid (SCFA)-producing bacteria, critically modulates gut antiviral immunity and the mucosal immune system (3, 4). It has been reported that *Akkermansia muciniphila*, or some genera, such as *Ruminococcus* and *Bifidobacterium*, which are SCFA-producers, are associated with improved clinical responses to immune checkpoint inhibitors (ICIs) therapy in cancer patients. This is correlated with an increased systemic immune tonus (5–8). These studies highlight the enormous potential of the gut microbiota in immunotherapy. Meanwhile, such gut microbiota also has significant potential in regulating the functions of immune cells to eliminate viruses (9, 10).

Despite advancements in vaccines, viral diseases remain a significant threat to both humans and animals due to rapid virus mutation (11). The rapid and continuous mutation of epidemic strains makes the prevention and control of viral diseases intractable. Antivirals and vaccines are unable to fully contain emerging and re-emerging viral epidemics. The mucosal barrier, which viruses must penetrate when infecting host cells, is colonized by a large number of bacteria. These bacteria have a symbiotic relationship with the host and regulate antiviral immunity through intricate and diverse pathways (2, 12). Viral infections and other factors (*e.g.*, antibiotic use) dynamically reshape the gut microbiota, thereby influencing viral disease outcomes (13–15). Sometimes, it is challenging to distinguish the cause-and-effect relationship between changes in the gut microbiota and the host’s susceptibility to viruses. It is crucial to properly understand the relationships between gut microbiota alterations and viral diseases and identify the constituents and effectors (*e.g.*, bacterial metabolites and components) of the gut bacterial microbiota (16).

Recently, SCFAs—primarily acetate, propionate, and butyrate—have garnered significant attention for their roles in regulating multiple human physiological systems, including the nervous, digestive, respiratory, cardiovascular, and immune systems, as well as their implications in tumor therapy (16–19). Among these, the immunomodulatory functions of SCFAs have been particularly well-documented. Specifically, gut microbiota-derived SCFAs emerge as key regulators of antiviral immunity, as evidenced by their protective roles in infections caused by influenza virus, respiratory syncytial virus (RSV), and porcine epidemic diarrhea virus (PEDV) (**Graphical Abstract**). These effects are mediated through diverse immune regulatory mechanisms, including but not limited to enhancing interferon responses, modulating inflammatory cytokine production, and maintaining epithelial barrier integrity (20–24). Given the growing understanding of these SCFA-mediated antiviral pathways, leveraging their therapeutic potential—particularly through novel probiotic-based interventions—holds promise for advancing viral disease containment strategies. The mechanisms underlying SCFA-mediated immune regulation have been continuously elucidated alongside technological advancements over the past decades, including metagenomics, untargeted metabolomics, and mass cytometry. In the present review, we aim to synthesize and discuss how gut microbiota regulates cellular antiviral functions, with a focused discussion of SCFAs’ roles in antiviral immunity—particularly those involving intracellular signaling pathways. This review will provide our insights into the antiviral mechanisms of gut microbiota and establish a foundation for therapies targeting microbiota modulation.

## 2 The profile of SCFAs

**Figure 1:**
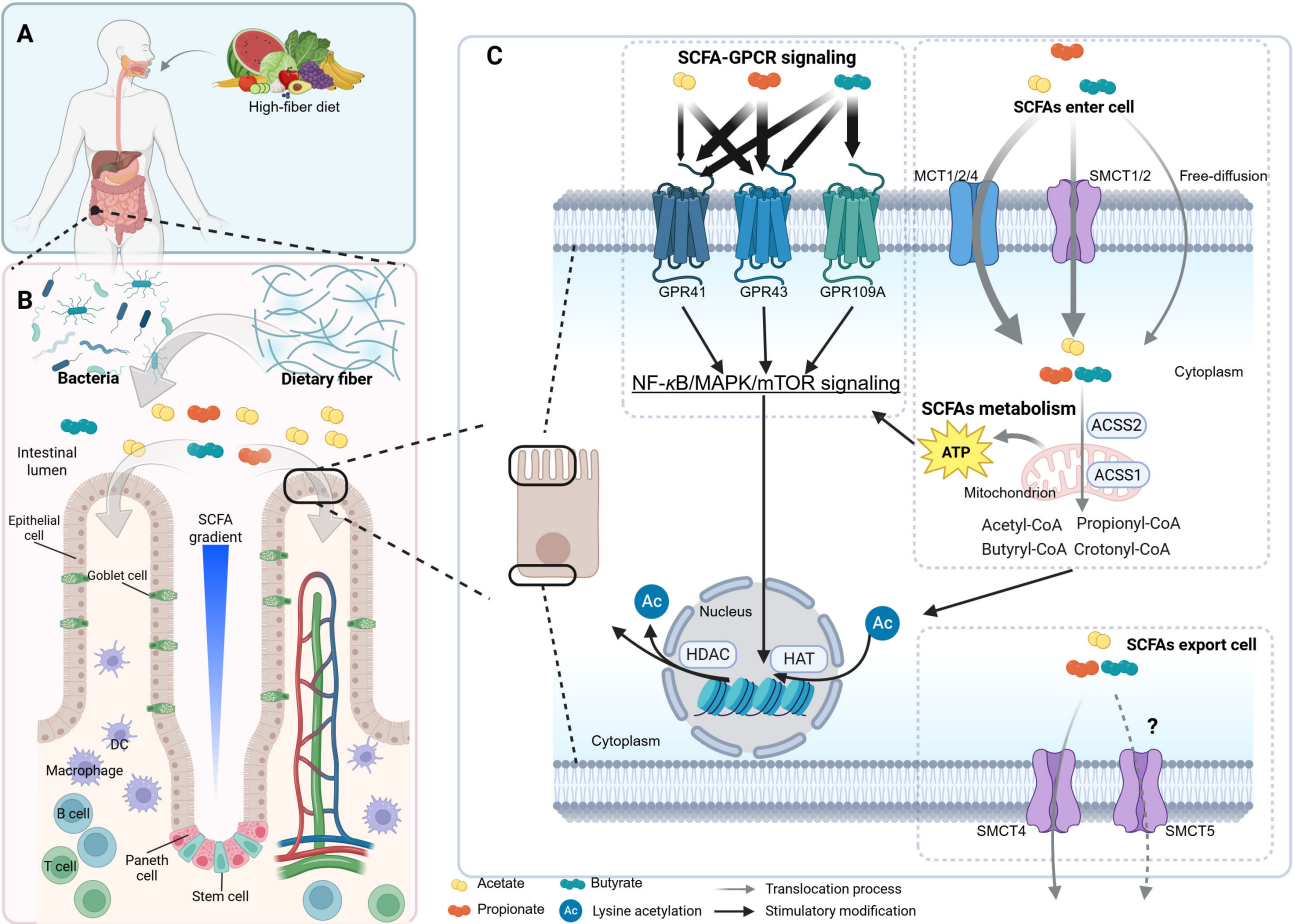
The profile of SCFAs. The scheme illustrates the general processes of short-chain fatty acid (SCFA) production, absorption, metabolism, and intracellular signaling pathways, along with their concentration gradient and cellular accessibility under physiological conditions. **(A, B)** A high-fiber diet promotes the expansion of SCFA-producing bacteria, which ferment dietary fiber in the intestinal lumen to generate SCFAs. These SCFAs form a concentration gradient that influences various cell types, including goblet cells, Paneth cells, and T cells. **(C)** Panel C profiles SCFA-GPCR signaling pathways and SCFA metabolism. SCFAs are enzymatically transformed into acyl-CoA by CoA synthases. For instance, acetate is converted to acetyl-CoA in the cytoplasm by acetyl-CoA synthetase 2 (ACSS2) or in mitochondria by ACSS1. For a more comprehensive understanding of the details related to SCFA metabolism, please refer to **Figure 4**. The tricarboxylic acid (TCA) cycle and glycolysis supply adenosine triphosphate (ATP) for the NF-*κ*B/MAPK/mTOR signaling and antiviral gene transcription. mTOR, mechanistic target of rapamycin. The figure was created using BioRender (https://BioRender.com).

SCFAs, mainly acetate, propionate, and butyrate, are primarily produced in the gut by fermenting dietary fiber and certain amino acids by gut microbiota, especially symbiotic bacteria (**Figures 1A, B**) (25). Some microbial communities like *Bacteroides* spp., *Blautia* spp., *etc.*, have been summarized in other reviews and are not further elaborated here (16, 26). The molar ratios of acetate, propionate, and butyrate in colonic contents are approximately 60–70: 20–30: 10–20 (**Figure 1B**) (27, 28). More than 90% of SCFAs are absorbed from the intestinal cavity and utilized as an energy source by colonocytes or liver cells (29, 30). SCFAs provide 60–70% of the energy supply for colonocytes, and butyrate is a primary energy source for them (31). The SCFAs not metabolized by colonocytes reach the liver *via* the portal vein. Among these SCFAs, butyrate and propionate are almost entirely taken up by the liver with normal hepatic functions (27, 29, 32). In peripheral blood, the majority of SCFAs is acetate (33, 34), and the molar ratio of acetate, propionate and butyrate in human peripheral blood is 91: 5: 4, as described previously (27).

SCFAs modulate cellular physiology by binding to and activating SCFA-sensing G-protein-coupled receptors (GPCRs), commonly referred to as SCFA receptors (SCFARs). This mechanism is discussed in detail in Section 3. Generally, SCFAs enter cells through primary mechanisms: (1) active transport *via* the monocarboxylate transporters (MCT1, MCT2, and MCT4) in relatively large amounts and the sodium-coupled monocarboxylate transporters (SMCT1, a high-affinity transporter, and SMCT2, a low-affinity transporter) in relatively smaller amounts; (2) passive diffusion (35–38). Moreover, after SCFAs are metabolized within cells, the remaining SCFAs are likely transported out of the cell across the basolateral membrane *via* the SCFA^-^HCO3^-^ antiport and the cation-SCFA anion symport. It is suggested that MCT4 or MCT5, both H^+^-dependent transporters, are responsible for these processes (**Figure 1C**) (39, 40). However, direct evidence for whether MCT5 can transport SCFAs is still lacking. SCFAs (*e.g.*, butyrate) inhibit histone deacetylases (HDACs), thereby modulating histone acetylation by regulating the balance between histone acetyltransferases (HATs) and HDACs (**Figure 1C**). This affects transcription by targeting distinct HDAC isoforms and further modulates cell functions (26, 41), as detailed in Section 4.

## 3 The molecular mechanisms of SCFA-GPCR signaling pathways and their implications on host antiviral immunity

### 3.1 Fundamental concepts and importance of SCFARs

Upon reaching the cell periphery, SCFAs bind to and activate SCFARs, a subclass of GPCRs, which are the most abundant membrane protein family in mammals, with over 800 members identified (42). To date, three main types of well-characterized SCFARs have been identified: G-protein-coupled receptor 41 (GPR41) (also known as free fatty acid receptor 3, FFAR3), GPR43 (free fatty acid receptor 2, FFAR2), and GPR109A (hydroxycarboxylic acid receptor 2, HCAR2). These receptors exhibit distinct ligand preferences: acetate preferentially activates GPR43, while propionate activates both GPR43 and GPR41, and butyrate shows higher affinity for GPR109A than GPR41 (**Figure 1C**, **Table 1**) (26, 43, 44). SCFARs are differentially expressed in immune, endocrine, and epithelial cells, where they play a central role in regulating cellular metabolism in both humans and mice (**Table 1**). Accumulating evidence highlights the critical roles of SCFAs and SCFARs in host physiology and tumor suppression, particularly in colorectal cancer (45–47). Recent studies have also implicated the SCFA-GPCR axis in modulating inflammation and antiviral immunity (15, 22). However, the precise molecular mechanisms underlying receptor activation in different cell types remain incompletely understood.

**Table 1:** Receptors of SCFAs.

SCFA receptors	SCFA affinity	G*α* subunits	Source animals	Tissue distribution	References
GPR41 (FFAR3)	Propionate > butyrate > acetate	G*α*i/o	Human, mouse, swine	Intestine, lymph nodes, neuron, bone marrow, spleen, lung, adipose, breast, pancreas, renal tubule; L cells, dendritic cells (DCs), peripheral blood mononuclear cells, and polymorphonuclear cells.	(43, 45, 219–225)
GPR43 (FFAR2)	Propionate ≥ acetate ≥ butyrate	G*α*i/o, G*α*q	Human, mouse, swine	Intestine, spleen, bone marrow, polymorphonuclear neutrophils (PMNs), pancreas, renal tubule; L cells, peripheral blood mononuclear cells, and polymorphonuclear cells.	(43, 45, 219–225)
GPR109A (HCAR2)	Only butyrate	G*α*i/o	Human, mouse	Colon, small intestine, retinal pigment epithelia; intestinal epithelial cells, colon cancer cell lines, adipocytes, monocytes, macrophages, neutrophils, DCs, and epidermal Langerhans cells.	(44, 46, 226)

This table summarizes the information of SCFA receptors, including their aliases, SCFA affinity, associated G*α* subunits, source animals, tissue distribution, and references.

### 3.2 Structure and signaling of GPCRs

Since the SCFARs belong to the GPCR family, we provide a brief overview of the structural features and signal transduction mechanisms common to GPCRs. Structurally, all GPCRs are characterized by seven transmembrane *α*-helical domains, separated by alternating intracellular and extracellular loops (42). Despite sharing these structural and activation mechanisms, GPCRs typically interact with specific heterotrimeric G proteins, which consist of *α*, *β*, and *γ* subunits (48). GPCRs exhibit diverse signaling outputs, with individual receptors activating distinct combinations of G-protein-dependent and G-protein-independent pathways.

**Figure 2:**
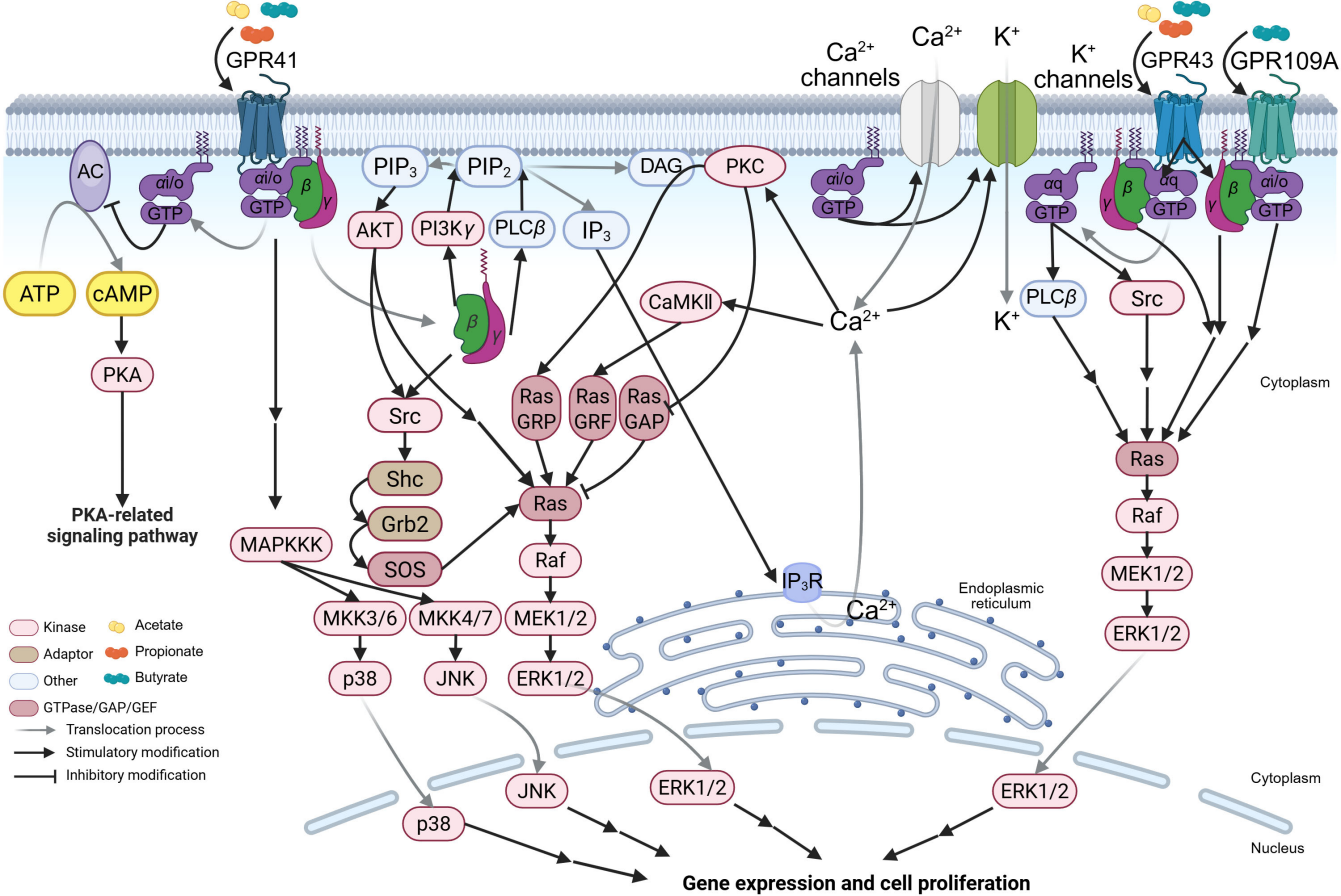
The SCFA-GPCR-G-protein signaling pathways. The schematic illustrates the detailed molecular mechanisms by which acetate, propionate, and butyrate activate short-chain fatty acid (SCFA)-sensing G-protein-coupled receptors (GPCRs) (including GPR41, GPR43, and GPR109A) and signal through distinct heterotrimeric G proteins, thereby initiating the mitogen-activated protein kinase (MAPK) signaling pathway. MKK, MAPK kinase; MAPKKK, MAPK kinase kinase; Ras, rat sarcoma viral oncogene homolog; Raf, rapidly accelerated fibrosarcoma; Ras GAP, Ras GTPase activating protein; JNK, c-Jun-NH_2_-terminal kinase; MEK, mitogen-activated protein kinase/extracellular signal-regulated kinase kinase; ERK, extracellular signal-regulated kinase. The figure was created using BioRender (https://BioRender.com).

The activation of canonical G-protein-dependent pathways in GPCRs follows a conserved mechanism: upon binding of an agonist (*e.g.*, SCFAs) to its corresponding receptor, the agonist-bound GPCR recruits and activates heterotrimeric G proteins. Acting as a guanine nucleotide exchange factor (GEF), the activated GPCR catalyzes GDP–GTP exchange on the G*α* subunits, inducing dissociation of GTP-bound G*α* from G*βγ* dimers (48). Based on sequence homology, G*α* subunits are classified into four families (G*α*s, G*α*i/o, G*α*q/11, and G*α*12/13) (49). Both GTP-G*α* and the G*βγ* dimer function as active signaling moieties, interacting with downstream effectors such as phospholipase C beta (PLC*β*), adenylyl cyclase (AC), and phosphatidylinositol 3-kinase (PI3K) (50, 51). These effectors subsequently propagate signals through pathways like Ras-ERK (**Figure 2**), ultimately regulating cellular metabolism and function. Signal termination occurs when GTP hydrolysis converts GTP-G*α* to GDP-G*α*, allowing both GDP-G*α* and G*βγ* to disengage from the downstream effectors and reassemble into inactive heterotrimers, resetting the receptor for subsequent stimulation (52).

**Figure 3:**
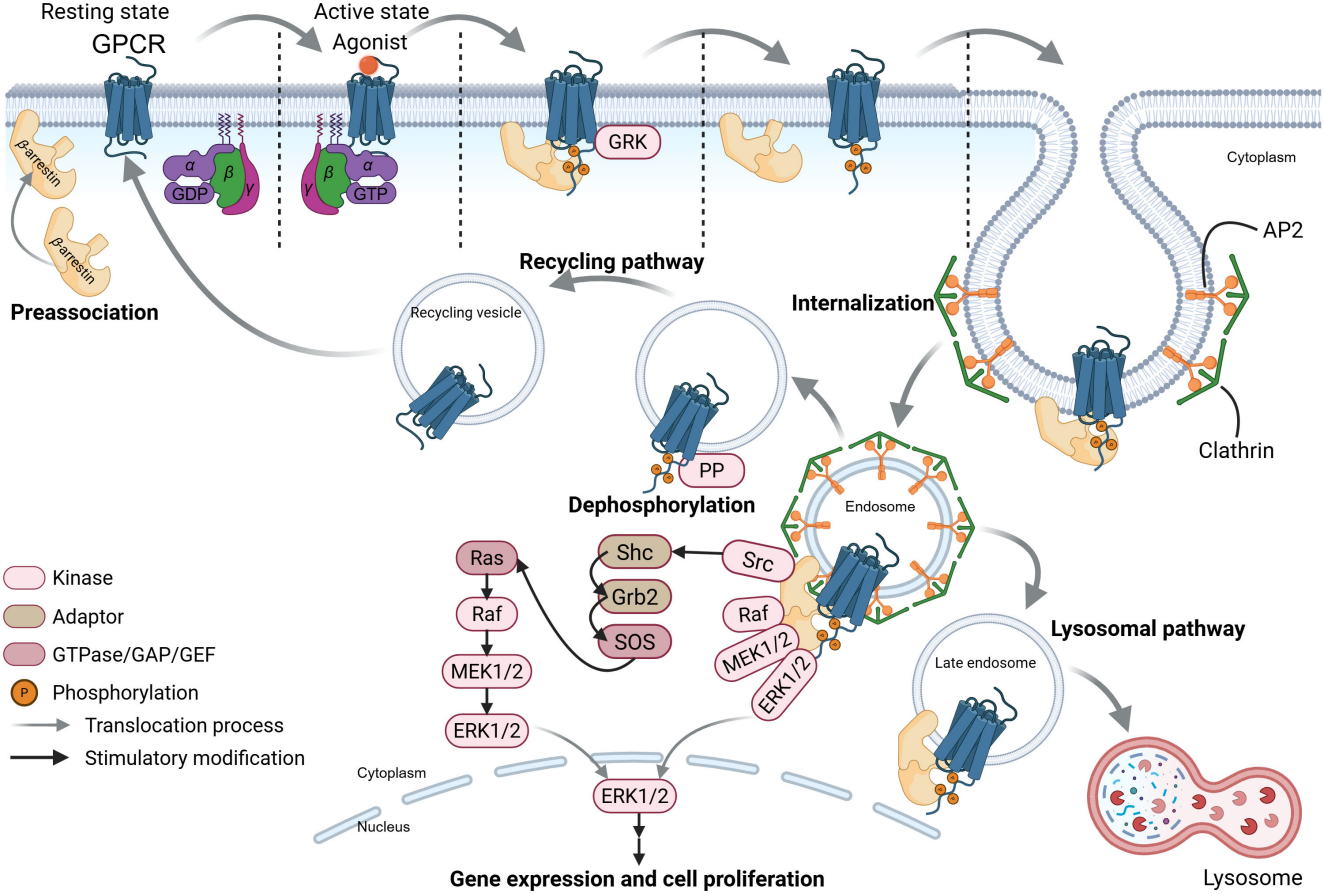
The SCFA-GPCR-*β*-arrestin signaling pathways. This schematic depicts ligand-induced G-protein-coupled receptors (GPCRs) recycling, desensitization, and *β*-arrestin-biased signaling pathways of these receptors. GRK, G-protein-coupled receptor kinase; AP2, adaptor protein 2; PP, protein phosphatase. The figure was created using BioRender (https://BioRender.com).

In parallel, G-protein-independent pathways are initiated through G-protein-coupled receptor kinase (GRK)-mediated phosphorylation and *β*-arrestin recruitment. Following agonist binding, the dissociated G protein activates downstream G-protein-dependent signaling, while GRKs phosphorylate the activated GPCR. This phosphorylation recruits plasma membrane-preassociated *β*-arrestin, which transiently couples to the receptor *via* lateral diffusion (53, 54). Membrane stabilization prolongs *β*-arrestin membrane-association, enabling its transient detachment from the activated GPCR and translocation to clathrin-coated pits (**Figure 3**) (54). *β*-arrestin then facilitates GPCR internalization by interacting with adaptor protein 2 (AP2) and clathrin heavy chain (55). Internalized GPCRs exhibit two fates: (1) dephosphorylation by protein phosphatases (PPs) in endosomes promotes recycling to the plasma membrane (52, 56, 57); (2) ubiquitination targets receptors to late endosomes for lysosomal degradation, a process termed “GPCR desensitization” (53, 58). Notably, *β*-arrestin bound to internalized GPCR-endosome complexes can also trigger signaling: it scaffolds Src family proteins and mitogen-activated protein kinase (MAPK) components (*e.g.*, Raf, MEK, ERK) to form a complex that activates the ERK pathway, influencing cell proliferation, differentiation, and survival (59–61). This *β*-arrestin-mediated, G-protein-independent signaling is termed “*β*-arrestin-biased signaling” (**Figure 3**). While these mechanisms were primarily characterized in *β*-adrenergic receptors, their conservation across GPCR subtypes—including SCFARs (GPR41, GPR43, GPR109A)—supports broad applicability.

The G-protein-dependent and -independent signaling pathways of GPCRs work coordinately in cellular signal transduction. Next, we detail the interaction networks among the signaling pathways.

### 3.3 Signaling characteristics of SCFARs

The differences in intracellular signal transduction among SCFARs primarily stem from receptor-G*α* coupling specificity and downstream G-protein-dependent pathways. Here, we use GPR41 as an example to detail its molecular mechanisms, with distinctions between GPR43/GPR109A presented separately. Key potencies of SCFAs in activating these receptors are summarized in **Table 1**.

GPR41 preferentially couples to the pertussis toxin-sensitive G*α*i/o family. Upon activation, G*α*i/o inhibits AC, reducing cyclic adenosine monophosphate (cAMP) synthesis (45, 62). Since AC converts adenosine triphosphate (ATP) to cAMP, this inhibition suppresses cAMP-dependent protein kinase A (PKA) activity (63), thereby dampening PKA-regulated pathways (*e.g.*, metabolism, gene expression) (**Figure 2**). The cellular outcomes vary by context: in human airway smooth muscle, GPR41 activation contracts tissue by reducing cAMP and elevating intracellular Ca^2+^ (64); in mouse pancreatic islets, it inhibits glucose-stimulated insulin secretion (65).

G*α*i/o also directly modulates ion channels: it prevents Ca^2+^ channel closure, promoting extracellular Ca^2+^ influx (66), and enhances K^+^ channel activity, inducing K^+^ influx that hyperpolarizes the membrane and lowers Ca^2+^ channel activation thresholds (67). These effects regulate Ca^2+^-dependent functions (*e.g.*, neurotransmitter release) (**Figure 2**) (68). Concurrently, dissociated G*βγ* activates PLC*β*, hydrolyzing phosphatidylinositol-4,5-diphosphate (PIP_2_) to generate inositol-1,4,5-trisphosphate (IP_3_) and diacylglycerol (DAG) (67, 69). IP_3_ triggers Ca^2+^ release from the endoplasmic reticulum *via* IP_3_ receptors (IP_3_R), elevating cytosolic Ca^2+^ (70, 71), which activates Ca^2+^-dependent kinases/calmodulin kinase II (CaMKII) and the Ras-ERK pathway (72–74). DAG, remaining membrane-bound, recruits protein kinase C (PKC) in a Ca^2+^-dependent manner to phosphorylate substrates like Ras guanyl-releasing protein (Ras GRP), further activating Ras-ERK (**Figure 2**) (72–74). Additionally, G*βγ* directly binds PI3K*γ* (highly expressed in leukocytes) (75, 76), converting PIP_2_ to phosphatidylinositol-3,4,5-trisphosphate (PIP_3_). PIP_3_ recruits and activates protein kinase B (AKT), indirectly promoting ERK1/2 activation *via* the Src family kinases/Shc/Grb2/SOS-mediated Ras-GTP conversion (77, 78). Activated Ras sequentially phosphorylates Raf–MEK–ERK1/2 (78). G*βγ* also directly binds and activates Src, initiating parallel Ras-ERK signaling (**Figure 2**) (79). Notably, PIP_3_ recruits PH domain-containing proteins to amplify Ras-ERK activation *via* Src (80). This G*βγ*-PI3K axis may regulate leukocyte migration during viral inflammation, facilitating viral clearance (81).

Unlike GPR41, GPR43 couples to both G*α*i/o and pertussis toxin-insensitive G*α*q (45). Thus, it activates both G*α*i/o-mediated pathways (above) and a distinct G*α*q-PLC*β* cascade: G*α*q-GTP hydrolyzes PIP_2_ into IP_3_/DAG (**Figure 2**) (69), triggering Ca^2+^/DAG-dependent signaling akin to G*βγ*. Furthermore, G*α*q-GTP directly binds to the Src SH3 domain, inducing conformational changes that activate Ras-ERK (**Figure 2**). Despite shared Ras-ERK outcomes, GPR41 and GPR43 diverge in molecular steps.

GPR109A primarily couples to G*α*i/o (like GPR41) and selectively binds butyrate among SCFAs (46, 82). Thus, its signaling closely resembles GPR41’s G*α*i/o-dependent pathways (**Figure 2**), though tissue-specific expression (**Table 1**) dictates functional differences. For example, GPR109A is upregulated in inflammatory bowel disease (IBD) patient epithelia and lamina propria macrophages (CD68^+^) (83). Silencing GPR109A in M1 macrophages reduces IL-1*β*/IL-6/TNF-*α* mRNA and secretion (83). SCFA-mediated GPCR activation (GPR41/GPR43) broadly modulates leukocyte functions, including cytokine production (TNF-*α*/IL-2/IL-6/IL-10) and migratory capacity (81, 84). These cytokines are critical for early viral clearance and antiviral immunity.

### 3.4 The roles of the MAPK signaling pathway in immune cells and the influence of SCFAs

Activation of SCFARs enhances antiviral immunity by modulating the MAPK signaling cascade, which regulates both coordinately adaptive and innate immune responses.

In T cells, MAPK pathways (ERK, JNK, and p38) are critical for activation, proliferation, and differentiation. Viral antigen stimulation triggers these pathways to prime T-cell effector functions and cytokine production—particularly interferon-gamma (IFN-*γ*)—thereby promoting antiviral adaptive immunity (85). ERK signaling predominantly enhances T-cell clonal expansion and survival (85, 86), while JNK and p38 govern differentiation and pro-inflammatory cytokine secretion, facilitating the generation of IFN-*γ*-producing Th1 cells essential for viral clearance (85–87). Consistent with the role of MAPK pathways in T cells, dietary SCFAs have been shown to enhance CD8^+^ T cell effector functions during influenza virus infection. Specifically, butyrate—a key diet-derived SCFA—alleviated excessive tissue damage caused by neutrophil infiltration and potentiated CD8^+^ T cell-intrinsic antiviral responses by reprogramming cellular metabolism in a GPR41 (FFAR3)-dependent manner (20). These findings underscore the critical role of GPR41 in mediating SCFA regulation of T cell responses during viral infections, as well as the impact of SCFA-induced metabolic reprogramming on antiviral immunity. Although Trompette et al. did not fully elucidate the role of the SCFA-GPCR-MAPK axis in this context (20), existing evidence strongly suggests its involvement (**Figure 2**).

Beyond T cells, SCFAs orchestrate innate immune cell responses to establish immune equilibrium. In allergic airway inflammation models, dietary SCFAs increased dendritic cell (DC) accumulation in the airways, which reduced allergen presentation to T cells and protected against pathology in a GPR41-dependent manner (88). Similarly, in influenza-infected mice, a high-fiber (HF) diet promoted the accumulation of alternatively activated macrophages that produced lower levels of CXCL1, thereby attenuating early neutrophil infiltration and tissue damage. This macrophage-driven suppression of excessive innate responses synergized with enhanced CD8^+^ T cell activity, ultimately improving viral clearance. Notably, oral butyrate administration alone was sufficient to confer protection against influenza, and this effect was dependent on GPR41 (20). These results highlight SCFAs’ ability to balance innate and adaptive immunity, resolving infections while preventing immunopathology.

Given the established role of MAPK pathways in macrophage and DC function (*e.g.*, TNF-*α*, IL-6, and type I IFN production) (89, 90), it is plausible that butyrate-induced GPR41 activation is engaged in MAPK signaling to amplify antiviral responses (**Figure 2**). This hypothesis aligns with evidence that p38 MAPK activation enhances macrophage phagocytic and virucidal capacities by stimulating inflammatory cytokine secretion, directly contributing to viral clearance (86).

Collectively, these findings position SCFAs as critical modulators of MAPK signaling in immune cells. SCFAs amplify MAPK activity, whereas deficiency of SCFARs abolishes their immunomodulatory effects (91, 92). Mechanistically, SCFAs alone do not induce significant MAPK phosphorylation in unstimulated cells; however, they potentiate cellular responsiveness to external stimuli (*e.g.*, viral infection), enabling rapid MAPK activation upon pathogen encounter (92, 93). These findings suggest that SCFAs may prime immune cells to maintain a heightened state of readiness (“pre-activated state”) or operate through an intrinsic mechanism that optimizes their “utilization mode” in response to infections. Future research should elucidate the more detailed intracellular molecular mechanisms underlying SCFAR activation-induced regulation of the MAPK pathway and its impact on immune cell function.

### 3.5 Multiple-ligand-coupling characteristics of GPCRs and research prospects

Although early studies proposed that individual GPCRs preferentially couple to a single G protein subtype (94), subsequent research has demonstrated that most GPCRs exhibit promiscuous coupling to multiple G proteins (95–100). This multi-valent coupling capacity likely underlies the remarkable ability of GPCRs to orchestrate complex cellular responses. Such coupling diversity is an inherent property of GPCRs, determined by specific amino-acid residues on the GPCR surface that mediate interactions with the G*α* subunit (95, 101). Notably, natural genetic variations in GPCRs—particularly missense mutations within G*α*-binding residues—frequently alter G*α* selectivity, with potential physiological consequences (101). Furthermore, signaling through distinct G proteins often occurs in a temporally regulated manner, enabling GPCRs to sequentially activate multiple signaling cascades (95, 102).

As members of the GPCR family, GPR41, GPR43, and GPR109A likely operate within this framework, promiscuously coupling with G*α*i/o and G*α*q to modulate cell physiology in response to diverse stimuli (**Figure 2**), including viral infection (45, 82). Although direct experimental evidence remains limited, this promiscuous coupling may contribute to the broad physiological effects of SCFAs. Future studies should investigate the mechanistic basis of G protein coupling promiscuity across SCFARs and the impact of epigenetic regulation on the spatiotemporal dynamics of G protein gene expression and their interaction with SCFARs.

In summary, GPCRs regulate cellular functions through an intricate network of positive and negative feedback loops (98), with SCFAs serving as key initiators of relevant GPCR pathways. However, critical aspects—such as the temporal dynamics of G-protein selectivity, the relative efficiencies of distinct SCFA-SCFAR and SCFAR-G-protein interactions, and their integrated physiological impacts—require further investigation.

## 4 The complex interactions and multifaceted functions of SCFAs and HDACs

### 4.1 The butyrate paradox: context-dependent dual effects of SCFAs on immune cells *via* HDAC inhibition and metabolic reprogramming

While SCFA-GPCR signaling pathways are critical for antiviral immunity, the interaction between SCFAs and HDACs significantly modulates the functions of immune effector cells. Notably, SCFA functions are highly dependent on their intracellular concentration profiles. Arpaia et al. demonstrated that butyrate and propionate promote extrathymic regulatory T cell (Treg) generation, with bacterial SCFAs—particularly butyrate—influencing the balance between pro- and anti-inflammatory pathways (103). These dual effects were linked to HDAC inhibition by butyrate and propionate, but not acetate.

Whitehead et al. first reported that butyrate suppresses proliferation and induces differentiation in colon carcinoma cell lines (104), a phenomenon replicated in other tumor-derived models (105–107). However, *in vivo* animal studies reveal a paradox: butyrate does not inhibit intestinal epithelial proliferation as observed in tumor cells; instead, it enhances epithelial renewal (108–110). Non-tumor colonic cell line experiments further show that butyrate can stimulate proliferation and suppress differentiation (111). This dichotomy—termed “the butyrate paradox”—highlights opposing effects of butyrate on healthy versus cancerous colonocytes or immune cells (103, 111, 112), extended to describe its context-dependent pro- or anti-inflammatory roles in immune cells.

**Figure 4:**
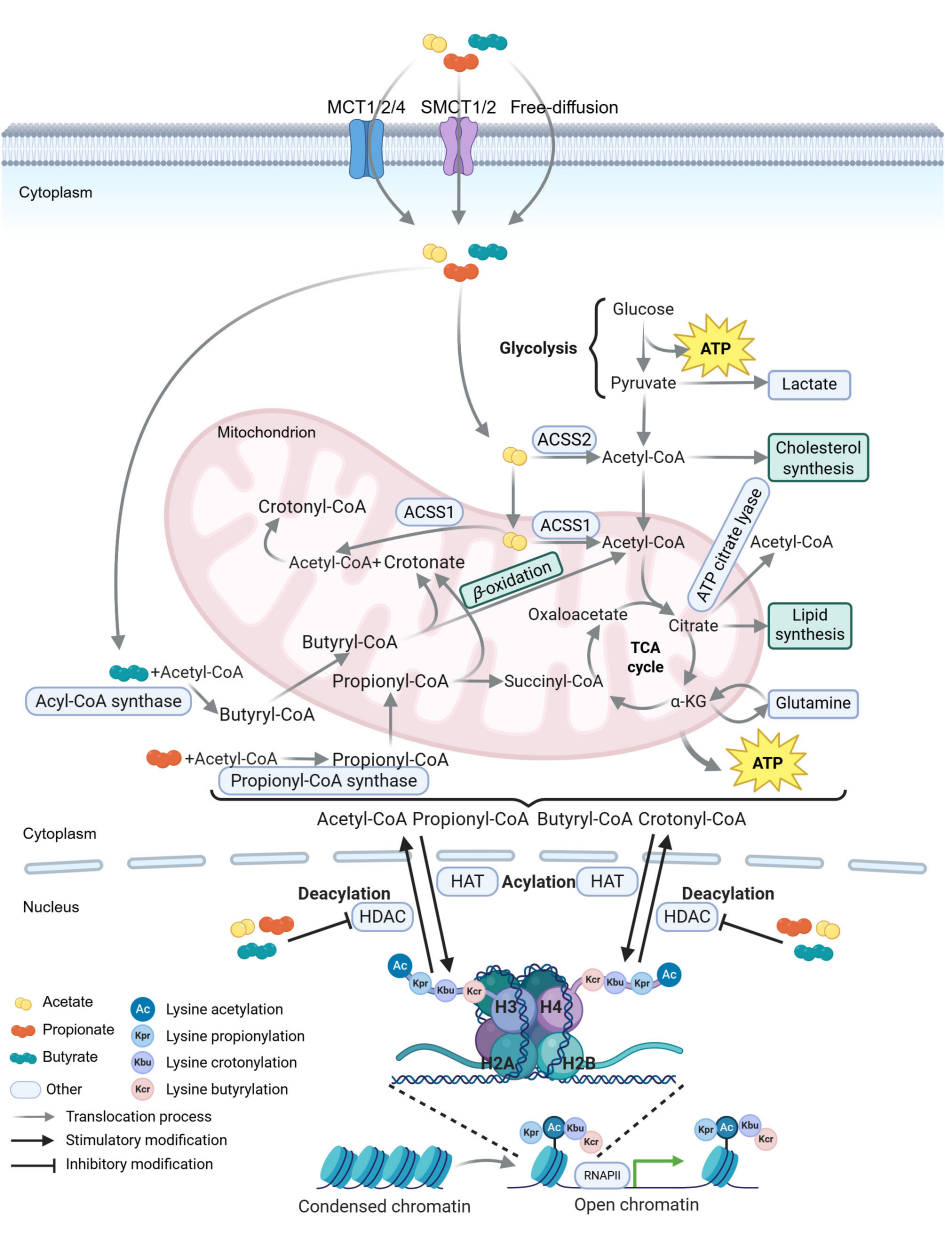
Schematic representation of SCFA metabolism and histone acylation equilibrium. Short-chain fatty acids (SCFAs) enter cells *via* monocarboxylate transporters (MCTs and SMCTs) or free diffusion. Acetate is converted to acetyl-CoA in the cytoplasm by acetyl-CoA synthetase 2 (ACSS2) or in mitochondria by ACSS1 (227, 228). Acetyl-CoA either enters the tricarboxylic acid (TCA) cycle or is converted to citrate. Citrate is exported from the mitochondria to the cytoplasm and metabolized to acetyl-CoA by ATP citrate lyase (229). Propionate is activated to propionyl-CoA by propionyl-CoA synthase in the cytoplasm, which is further converted to succinyl-CoA through multistep reactions (230, 231). Butyrate is ligated to CoA by acyl-CoA synthase to form butyryl-CoA, which undergoes *β*-oxidation to generate acetyl-CoA that enters the TCA cycle (232). In mitochondria, propionyl-CoA and butyryl-CoA are converted to crotonate, which reacts with acetyl-CoA to form crotonyl-CoA (231, 233, 234). These acyl-CoA species (acetyl-CoA, propionyl-CoA, butyryl-CoA, and crotonyl-CoA) derived from SCFA metabolism provide acyl groups for lysine acylation of histone and non-histone proteins (235). Specifically, histone deacetylation mediated by histone deacetylases (HDACs) condenses chromatin and represses gene transcription, whereas histone acetylation by histone acetyltransferases (HATs) loosens chromatin and promotes transcription. RNAPII, RNA polymerase II. The figure was created using BioRender (https://BioRender.com).

Salvi et al. proposed a mechanistic explanation: butyrate modulates chromatin structure *via* HDAC inhibition, altering cellular transcriptional phenotypes (113). In the absence of HDAC inhibitors (HDACis), HDACs deacetylate histones (*e.g.*, histone H3), enhancing DNA binding and repressing transcription (**Figure 4**). Butyrate and propionate are natural HDACis (103, 114), but their efficacy depends on cellular metabolism. Differentiated intestinal epithelial cells rapidly metabolize butyrate for energy, limiting its accumulation and HDAC inhibitory capacity (113). In contrast, colon cancer cells exhibit the Warburg effect, prioritizing glycolysis over oxidative phosphorylation (OXPHOS) and glucose over SCFAs (115, 116). This metabolic preference allows butyrate to accumulate, act as an HDACi, and arrest cell cycle progression *via* transcriptional reprogramming. Additionally, butyrate induces metabolic reprogramming in colorectal cancer cells—modulating enzymes like pyruvate kinase M2 and pyruvate dehydrogenase complex (117–119)—thereby reversing the Warburg effect and exerting anti-neoplastic effects. Notably, the Warburg effect also occurs in normally proliferating cells (120), though for distinct reasons than in cancer. Proliferative cells—including virus-infected cells and activated immune cells (*e.g.*, CD8^+^ T cells)—adapt metabolism to prioritize nutrient uptake for biosynthesis of viral components, effector molecules, or new cells (116, 121, 122).

Predicting SCFA functions requires integrating their production sites, concentration gradients, and cellular accessibility. SCFAs are predominantly generated in the colorectal lumen by commensal bacteria (**Figure 1B**). Colorectal epithelial cells preferentially utilize SCFAs (especially butyrate) over glucose (123), whereas cancerous colonocytes, intestinal stem cells, small intestinal enterocytes, and T cells show reduced butyrate utilization due to lower local concentrations and accessibility (**Figure 1B**) (113, 124, 125). These factors determine intracellular SCFA levels, HDAC inhibition efficacy, and ultimately, cellular outcomes.

Consistent with this framework, studies have demonstrated context-dependent effects of butyrate mediated by HDAC inhibition and cellular metabolic state: for example, in a dextran sulfate sodium salt-induced colitis model, butyrate alleviated colonic inflammation by suppressing HDAC8 to blunt the NF-*κ*B pathway (126). During influenza infection, SCFA supplementation tended to reduce intestinal inflammation and reverse barrier disruption, thereby attenuating bacterial enteric infections and enhancing survival in doubly infected animals (127). The anti-inflammatory properties of SCFAs also mitigated symptoms following viral infection (22, 128). However, conflicting evidence suggests that butyrate may enhance viral replication under certain conditions: it increased cellular susceptibility to influenza virus, reovirus, and human immunodeficiency virus (HIV) infections by suppressing specific IFN-stimulated genes (ISGs) in human and mouse cells, with HDAC inhibition likely contributing to this regulation (129). Similarly, Yin et al. found that butyrate promoted transmissible gastroenteritis virus (TGEV) infection by inhibiting class I HDACs, which downregulated retinoic acid-inducible gene I (RIG-I) expression. This suppression impaired mitochondrial antiviral-signaling protein (MAVS) aggregation, reduced type I IFN and ISG production, and facilitated TGEV replication in porcine intestinal epithelial cells (130).

Notably, these studies used supraphysiological butyrate concentrations (2.5–5 mM) (129, 130), which far exceed the human total serum SCFA concentration range (79–375 *μ*M) (27). In contrast, Wang et al. employed lower concentrations (1 mM acetate, propionate, butyrate) in cell experiments (128), suggesting that cellular butyrate levels critically influence outcomes during viral infection.

In brief, the butyrate paradox reflects a dynamic interplay between HDAC inhibition and metabolic competition: butyrate’s dual effects are dictated by its intracellular concentration, which is governed by cellular metabolic state (proliferative vs. differentiated) and SCFA accessibility. This framework not only resolves apparent contradictions in butyrate’s functions but also highlights the need to integrate metabolic and epigenetic perspectives in understanding SCFA-mediated regulation of immunity and epithelial homeostasis. Future studies should elucidate how physiological SCFA gradients are established *in vivo* and how pathogens or inflammation disrupt these gradients to modulate host responses.

### 4.2 Histone acylation by SCFAs: beyond acetylation and their multifaceted roles in cellular processes

In addition to the canonical lysine acetylation mentioned above, several types of short-chain lysine acylations on histones, such as crotonylation (Kcr), propionylation (Kpr), and butyrylation (Kbu), have been recently identified (131–133). These acylations are associated with cellular metabolism and gene transcription regulation, and their levels are modulated by the availability of SCFAs and their coenzyme A (CoA) adducts in the cell (134, 135). SCFAs can be intracellularly metabolized into crotonate (2-butenoate), a metabolic intermediate (136). Crotonate is then converted into crotonyl-CoA, which stimulates gene transcription through p300-catalyzed histone crotonylation at sites such as H4K5, H4K12, H3K14, and H3K18 (**Figure 4**) (133, 134). Alternatively, SCFAs (such as butyrate) and crotonate inhibit class I HDACs (mainly HDAC1-3), the major enzymes responsible for histone decrotonylation (the removal of crotonyl groups from histones). The crotonylation at lysine 18 of histone H3 (H3K18cr) is associated with transcription start site (TSS). This modification is enriched in various pathways in colon epithelial crypts, including those related to cancer, adherens junctions, and the transforming growth factor-beta (TGF-*β*) signaling pathway, indirectly indicating that these genes are in a relatively active expression state. Cancer-related pathways typically involve processes such as cell proliferation and differentiation, which require high-level gene expression for maintenance (133). This is consistent with the experimental finding reported by Peng et al. that SCFA treatment can maintain the integrity of the intestinal epithelial barrier and promote the proliferation of intestinal epithelial cells (126).

Consistent with their role in modulating histone acylation, SCFAs have been implicated in regulating antiviral immune responses through both HDAC-dependent and -independent mechanisms: during influenza virus infection, Nagesh et al. demonstrated that the virus dysregulated HDAC1—a coactivator of the type I IFN response which normally inhibits viral replication—and that inhibition of HDAC1 activity increased influenza A virus (IAV) infection in a dose-dependent manner (137). This suggests that HDAC inhibition-induced histone acylation (*e.g.*, crotonylation) may be critical for activating the type I IFN response to counteract viral infection. Furthermore, histone crotonylation has been shown to reshape local chromatin architecture at the HIV long terminal repeat (LTR) *via* acetyl-CoA synthetase 2 (ACSS2)-mediated mechanisms, simultaneously reducing histone methylation. Notably, ACSS2 induction exhibited strong synergy with either a protein kinase C agonist (PEP005) or an HDACi (vorinostat) in reactivating latent HIV (138). These findings highlight specific chromatin sites of histone acylation as potential therapeutic targets for viral eradication strategies. Although the regulatory effects of SCFAs, HATs, and HDACs on histone crotonylation during viral infections remain incompletely characterized, their involvement is predicted by existing evidence.

Furthermore, emerging evidence suggests that SCFAs may directly modulate histone acylation independent of HDAC inhibition, thereby influencing viral latency and type I IFN responses: Nshanian et al. proposed a unique mechanism whereby SCFAs directly acylate lysine residues on histones in specific genomic regions, exerting antiproliferative effects in colorectal cancer (CRC) cells while promoting normal cell proliferation (CCD841 cells) without relying on HDAC inhibition (139). Specifically, propionate and butyrate are converted into propionyl-CoA and butyryl-CoA (**Figure 4**), which serve as cofactors for major HAT families to catalyze histone acylation with similar efficiencies (139–141). These CoA derivatives facilitate acylation at H3K18 and H4K12 (*e.g.*, H3K18pr, H3K18bu, H4K12pr, H4K12bu) in CRC cells, leading to homeostatic dysregulation through hyperactivation of the Wnt/*β*-catenin and TGF-*β* signaling pathways, oncogene activation (*e.g.*, *MYC*, *FOS*, *JUN*), and increased chromatin accessibility. Consequently, proto-oncogenes involved in growth and differentiation are further overexpressed, potentially triggering apoptosis—particularly under elevated butyrate conditions (139). Conversely, in normal cells, propionate was found to enhance epithelial homeostatic gene-expression pathways without significant HDAC inhibition-mediated acetylation (139). While dynamic molecular-pathway evidence is needed to fully elucidate this model, it broadens the understanding of SCFAs as direct epigenetic regulators—not merely HDAC inhibitors—thereby enriching the theoretical framework of the “butyrate paradox.”

As discussed above, SCFAs serve as critical regulators of histone acylation and HDAC inhibition (under higher intracellular concentrations, Section 4.1), deeply integrating into the balance between HAT and HDAC activities shaped by specific cellular metabolic states. This integration likely plays a pivotal role in regulating viral latency and type I IFN responses during viral infections. Nevertheless, the acylation-mediated effects of SCFAs in antiviral immunity remain to be fully elucidated.

### 4.3 The complex interplay between SCFAs, HDACs, and NF-*κ*B signaling in antiviral immunity and cellular regulation

**Figure 5:**
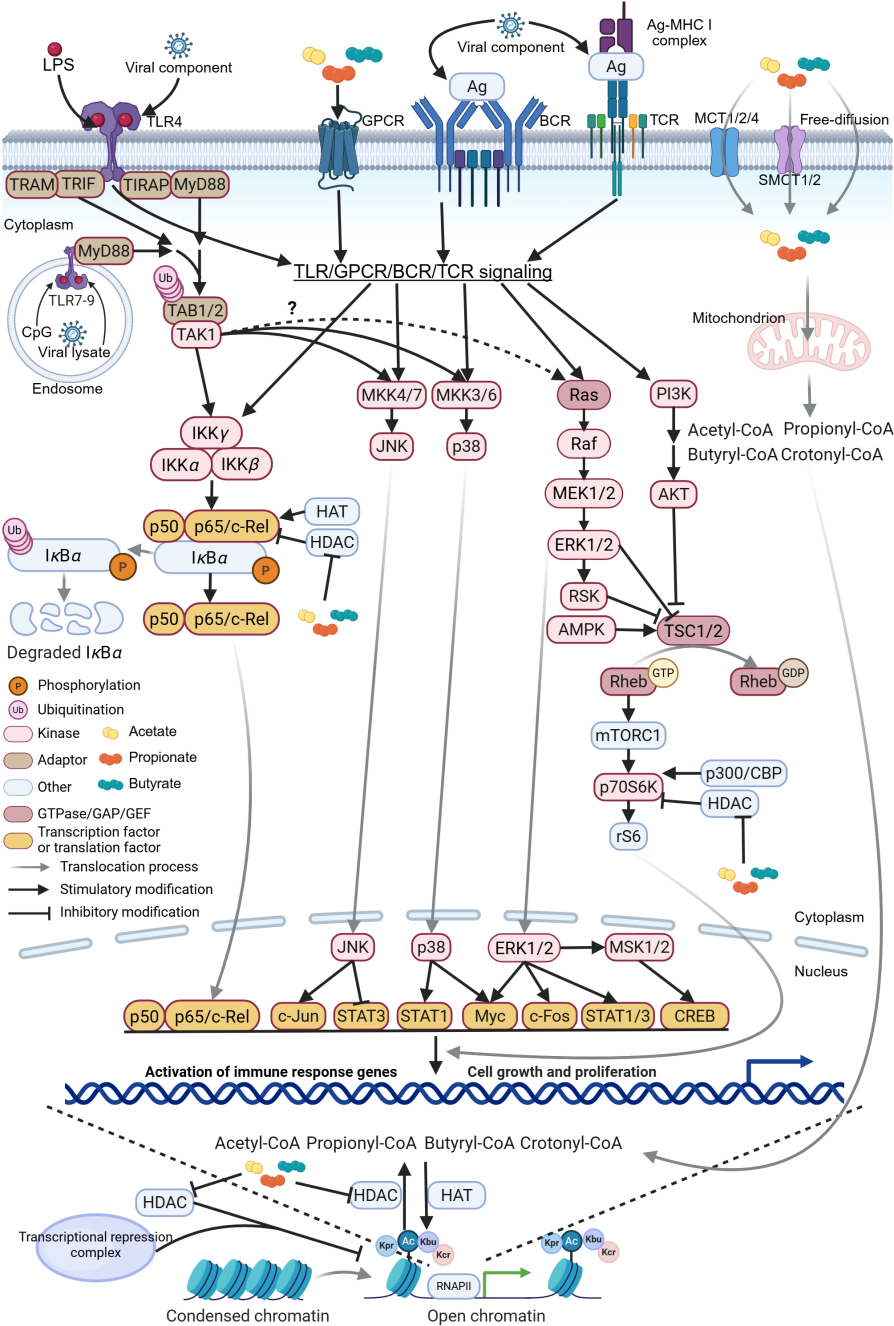
Schematic representation of SCFA-HDAC-HAT interaction on intracellular signaling pathways. The schematic illustrates the molecular mechanisms by which short-chain fatty acids (SCFAs), histone deacetylases (HDACs), and histone acetyltransferases (HATs) modulate toll-like receptor (TLR), G-protein-coupled receptor (GPCR), B-cell receptor (BCR), and T-cell receptor (TCR) signaling pathways. Upon activation, these receptor cascades converge to regulate downstream networks, including NF-*κ*B, JNK, p38, ERK, and mTOR, in a cell-context-specific manner. Notably, activated transforming growth factor *β*-activated kinase 1 (TAK1) contributes to the ERK signaling through a less characterized mechanism, potentially involving cross-talk with Raf kinases (236). Collectively, these pathways orchestrate effector T and B cell differentiation and antiviral immunity. RSK, ribosomal S6 kinase; AMPK, AMP-activated protein kinase; TSC, tuberous sclerosis complex; Rheb, Ras homolog enriched in brain; mTORC1, mammalian target of rapamycin complex 1; CREB, cAMP response element-binding protein; MSK, mitogen- and stress-activated protein kinase; STAT, signal transducer and activator of transcription; TRC, transcriptional repression complex. The figure was created using BioRender (https://BioRender.com).

The interactions between SCFAs and HDACs significantly influence the antiviral functions of immune effector cells (26). HDACs possess the capacity to remove acetyl moiety from acetyl-lysine residues on both histone and non-histone proteins (142). For instance, HDAC3-mediated deacetylation of NF-*κ*B subunits (*e.g.*, p65/RelA) stabilizes their association with I*κ*B*α*, thereby inhibiting NF-*κ*B transcriptional activity (**Figure 5**) (143, 144). HDACs can also bind to transcriptional repression complexes (TRCs) (*e.g.*, Sin3A and NuRD) and recruits these complexes to the promoter regions of the NF-*κ*B-regulated genes to suppress transcription. In ovarian cancer models, AT-rich interaction domain 1A (ARID1A) mutations impair HDAC complex recruitment, relieve NF-*κ*B inhibition, and activate pro-inflammatory cytokine expression (145). By deacetylating histones, HDACs condense chromatin structure, blocking NF-*κ*B binding to target gene promoters and reducing transcription of inflammation-related genes (*e.g.*, *il-6*, *tnf*). Notably, in head and neck squamous cell carcinoma, NF-*κ*B activation induces HDAC-mediated histone deacetylation, leading to chromatin compaction and reduced DNA repair capacity, which enhances chemotherapy resistance (146). Collectively, under physiological conditions, HDACs suppress the NF-*κ*B signaling pathway, thereby decreasing the expression of antiviral genes, including *ifnb1* and ISGs (147). This reduction in gene expression facilitates viral replication within host cells.

However, some studies suggest that HDAC3 is necessary for NF-*κ*B-dependent gene expression under special conditions (148, 149). This might be attributed to the selective deacetylation of specific NF-*κ*B subunit sites and genomic sites by HDAC3 (150). Moreover, the direct deacetylation of histone proteins, the important component of chromatin, by HDAC profoundly affects gene transcription (151). For instance, the tumor suppressor retinoblastoma (Rb) selectively binds to the *ifnb* enhancer region *in vivo* by interacting with c-Jun, a component of the IFN-*β* enhanceosome. Subsequently, Rb recruits HDAC1 and HDAC8, leading to a decrease in the acetylation of histone H3/H4 in the *ifnb* promoter and thus suppressing *ifnb* transcription (152). Chen et al. discovered that influenza A viruses (IAVs) utilize host HDAC1 to downregulate the acetylation level of NP. Specifically, the deacetylation at lysine 103 of NP promotes the replication efficiency of IAVs (153). Williams et al. demonstrated that NF-*κ*B p50-HDAC1 complexes constitutively bind to the latent HIV LTR, inducing histone deacetylation and repressive changes in the chromatin structure of the HIV LTR. This impairs the recruitment of RNA polymerase II and transcriptional initiation, thereby promoting HIV latency (154).

Notably, Adam et al. and Peng et al. found that HDACis can effectively inhibit NF-*κ*B activation (126, 155). Peng et al. reported that in human colon cancer Caco-2BBe cells, HDAC8 is essential for NF-*κ*B activation, which downregulates solute carrier family 26 member 3 (Slc26a3) and tight junction (TJ) proteins like ZO-1, occludin-1, and claudin-1. Butyrate and other HDACis were found to greatly reduce NF-*κ*B activation triggered by lipopolysaccharide (LPS) in Caco-2BBe cells by the decrease in the ratio of phosphorylated p65 (p-p65) to p65 and phosphorylated I*κ*B*α* (p-I*κ*B*α*) to I*κ*B*α*. The research findings of Peng et al. suggest that butyrate exerts its effect mainly by suppressing HDAC8 activity. By inhibiting HDAC8, butyrate attenuates the NF-*κ*B signaling pathways, thereby upregulating the expression of Slc26a3, which have been verified *in vivo* and is crucial for maintaining the integrity and function of the intestinal epithelial barrier (126).

Furthermore, this regulatory mechanism may also play a role in defending against viral invasion. Since the intestinal epithelial barrier acts as the first line of defense against pathogens, the enhancement of its function by butyrate through the modulation of the HDAC8-NF-*κ*B-Slc26a3 axis might potentially impede the invasion of certain viruses. For instance, rotavirus and PEDV infect intestinal epithelial cells by breaching the intestinal epithelial barrier. Moreover, the supplementation of SCFAs has been associated with a reduction in viral load *in vivo* (21, 156, 157). However, the specific mechanisms through which the antiviral effect of SCFAs occurs remain to be fully elucidated, and further research is warranted to explore their potential as a therapeutic target against viral infections.

### 4.4 The regulatory effects of SCFAs and HDACs on immune cell differentiation and related signaling pathways in antiviral immunity

HDACs also engage in interactions with key proteins of other intracellular signal pathways to modulate the functions of immune cells, including B cells and T cells. Meanwhile, SCFAs are involved in these regulatory processes. As we know, anti-CD40 monoclonal antibody (mAb), CpG, and LPS signal through BCR, toll-like receptor 9 (TLR9), and TLR4, respectively (**Figure 5**), inducing B cells to differentiate into B10 cells (158–160). When stimulated by anti-CD40 mAb, CpG, or LPS, SCFAs, especially butyrate, can significantly increase the production ratio of mouse and human B10 cells. Moreover, butyrate functions as an HDACi and its actions are independent of GPCRs activity. Both the treatment with butyrate and the HDACi vorinostat enhance the activity of ERK (p-ERK/ERK) and p38 (p-p38/p38). Meanwhile, they inhibit the activity of JNK (p-JNK/JNK), which contributes to the high-efficiency induction of B10 cells (161). B10 cells are regulatory B cells capable of producing IL-10 and contribute to the maintenance of the immune homeostasis and immunological tolerance.

Additionally, HDACs and p300/CBP (CREB-binding protein, a protein with HAT activity) modulate the acetylation status of p70 S6 kinase (S6K). S6K phosphorylates ribosomal protein S6 (rS6), and this process modulates the mTOR-S6K pathway (**Figure 5**) (162, 163), which determines the differentiation direction of T cells. Park et al. found that SCFAs, such as acetate and propionate, could enhance the acetylation of S6K and the phosphorylation of rS6 as inhibitors of HDACs (164). Subsequently, they further affect gene transcription in T cells through the mTOR-S6K pathway. Ultimately, this induces T cells to differentiate into effector T cells, including T helper type 1 (Th1) cells and T helper type 17 (Th17) cells, as well as IL-10^+^ Tregs, depending on the specific cytokine milieu and immunological context. Moreover, butyrate may act through the same pathway as acetate and propionate. This is because it similarly enhances the generation of Th1, Th17, and IL-10^+^ Tregs under all T-cell polarization conditions tested respectively (164).

However, these studies only reveal the phenomenon in different cells treated with SCFAs and do not fully elucidate the detailed and intricate molecular interactions. For example, SCFAs and HDACs do not directly participate in the phosphorylation of ERK and p38 as MAPK substrates or phosphorylases. ERK and p38 are protein kinases that phosphorylate their target proteins in specific signaling pathways, such as theTLR-MyD88-STAT3 pathway in B cells (165). But the treatment of SCFAs increases the phosphorylation of ERK and p38 (161). Thus, it is inferred that there should be other molecule(s) mediating this phosphorylation process. In the induced differentiation of T cells, SCFAs lead to enhanced phosphorylation of STAT3 and rS6 (164). This may be an indirect result of the inhibition of HDACs by SCFAs. Additionally, the epigenetic changes due to HDACs inhibition were not comprehensively considered in the above-mentioned studies.

Notably, SCFAs enhance B and T cell differentiation during active immune responses, but not under homeostatic conditions (161, 164). This may suggest that the functions of SCFAs in these cells are related to the cell state. Fundamentally, SCFAs function by involving intracellular metabolism and epigenetic processes that vary with different stimuli, such as viral infection. This could explain why the functions of SCFAs vary depending on the cell state. Furthermore, the intracellular signal pathways related to the differentiation of B cells and T cells, including the NF-*κ*B, TLR-MyD88-MAPK, and mTOR-S6K signaling pathways, are usually activated by viral infection. For example, vesicular stomatitis virus glycoprotein G activates the TLR4-TRIF-IRF7 pathway, which leads to a type I IFN response in myeloid DCs (mDCs) and macrophages rather than plasmacytoid DCs (166). Mouse mammary tumor virus (MMTV) infects and activates B cells through TLR4 in mice and also increases NF-*κ*B activity (167). In addition, viral components (such as viral nucleic acids and degradation products) are stimuli for TLR3, TLR4, TLR7, TLR8, and TLR9. The activation of these TLRs signals through downstream proteins such as NF-*κ*B, JNK, ERK, and p38 to induce immune responses and cell differentiation (**Figure 5**). This process could enhance the antiviral immunity (165, 168). Moreover, butyrate directly suppresses the activity of HDAC in antitumor cytotoxic CD8^+^ T cells. Subsequently, it enhances CD8^+^ T cell responses both *in vitro* and *in vivo* in an ID2-dependent manner by promoting IL-12 production. This indicates that butyrate may enhance the antitumor therapeutic efficacy through gene-transcriptional regulation of CD8^+^ T cells (122). This effect may also play a role in viral infections.

In summary, under normal cellular conditions, HDACs play a crucial role in cellular epigenetics by deacetylation modifications on both histone and non-histone proteins, such as by suppressing the production of inflammatory cytokines. Nevertheless, in the absence of HDACis, some viruses exploit this mechanism to enhance their own replication. Conversely, HDACis, such as trichostatin A (TSA), butyrate, and propionate, can counteract the effects of HDACs. Butyrate can inhibit HDACs by competitively binding to their active sites, as it has a similar structure to TSA and the acetyl-lysine on histones (169). The effects of butyrate and propionate still operate within the framework of the butyrate paradox mentioned above.

## 5 The composite role of SCFAs in antiviral immunity

### 5.1 The antiviral mechanisms of SCFAs in innate immunity: insights from acetate’s effects on respiratory syncytial virus infection

Building upon the previously discussed molecular mechanisms of SCFAs in intracellular actions, here we turn to specific examples to discuss the potential of SCFAs in modulating antiviral immunity. SCFAs regulate immunity and inflammatory responses in the gut and systemically (170, 171). Emerging evidence suggests they also directly influence the outcomes of viral infectious diseases through complex mechanisms. A HF diet promotes the growth of *Lachnospiraceae* family members, which are responsible for acetate production in the gut (22). Elevated acetate levels were shown to inhibit RSV replication in a manner dependent on type I interferon receptor (IFNAR) and GPR43, mediating IFN-*β* responses and upregulating ISGs in pulmonary epithelial cells (22). IFN-*β* is produced early during viral infection and acts through IFNAR to initiate inflammatory cytokine cascades critical for limiting viral replication and directing immune responses (172, 173).

While the precise mechanisms by which acetate modulates type I IFN production *via* IFNAR and GPR43 activation remain incompletely understood, one plausible hypothesis posits that acetate may help pulmonary epithelial cells overcome RSV NS protein-mediated inhibition of the type I IFN signaling pathway. This restoration of type I IFN production following viral invasion could involve GPR43 activation and acetylation of histones by acetate, which regulates inflammatory cytokine signaling pathways (*e.g.*, NF-*κ*B and Ras-ERK pathways) to prevent hyperinflammation (26). For instance, Xu et al. demonstrated that inflammation-induced *β*-arrestin1 promotes phosphorylation of NF-*κ*B p65 (174). GPR43 activation may redirect *β*-arrestin toward alternative functions, such as GPCR desensitization and Ras-ERK pathway activation (**Figure 3**), thereby relatively reducing *β*-arrestin-mediated p65 phosphorylation. Furthermore, GPR43 activation initiates the Ras-ERK signaling cascade, which orchestrates cytokine production and apoptotic responses critical for viral clearance (86). Viral components (*e.g.*, RNA) trigger TLR signaling, which converges with these pathways to amplify NF-*κ*B activation and IFN production (**Figure 5**). Additionally, virus-induced IFN-*β* aided by acetate further enhances transcription of well-characterized antiviral ISGs *Isg15* (encoding ISG15) and *Oas1* (encoding oligoadenylate synthetase) in pulmonary epithelial cells (22, 175). Oligoadenylate synthetase (OAS) synthesizes 2’-5’-oligoadenylate (2-5A), which induces viral RNA degradation *via* RNase L activation and directly inhibits viral replication (176, 177).

Consistent with these findings, microbiota-derived SCFAs significantly alleviate RSV-induced pathological damage and enhance host clearance of the virus, even though oral acetate treatment alone does not prevent RSV infection (22). This suggests the potential clinical value of acetate as a therapeutic agent. However, critical gaps remain in understanding the spatiotemporal dynamics of molecular interactions following acetate-GPR43 activation, as well as the coordinated regulation among the acetate-GPR43-MAPK, NF-*κ*B, and type I IFN pathways. Addressing these questions is essential to fully elucidate SCFAs’ antiviral mechanisms.

Furthermore, propionate and butyrate also exhibit robust RSV replication inhibitory effects (22), likely through distinct immune cell activation mechanisms that warrant further investigation. Collectively, SCFAs serve as critical mediators of intracellular signaling, enhancing cellular function and strengthening the innate immune response to viral invasion.

### 5.2 The role of SCFAs in modulating CD8^+^ T cell metabolism and function during viral infections

SCFAs not only exert effector functions in innate immunity but also act as critical regulators of adaptive immunity by engaging cellular metabolism. SCFAs, particularly in intestinal cells, are well-established energy substrates (30, 34). Recent studies have highlighted their roles in adaptive immune cells, including CD8^+^ T cells (20, 178). Microbiota-derived SCFAs serve as precursors for intracellular acetyl-CoA, fueling OXPHOS and glycolysis to meet the energy demands of CD8^+^ T cell immune responses during viral infections (**Figure 4**) (15, 20). These responses—including viral antigen recognition, effector cell differentiation, and transcription of antiviral genes and enzymes—require rapid energy production when viruses invade the host (15, 178).

In virus-infected tissues, immune cells often rely on glycolysis as a compensatory pathway when aerobic respiration is insufficient to meet energy demands (26, 179). Energy-related metabolic pathways and metabolites fundamentally influence lymphocyte differentiation, function, and fate (179). SCFAs have been implicated in multiple such pathways (**Figure 4**). For example, microbiota-derived acetate enhances antiviral responses and restores IFN-*γ* production in mucosal and peripheral CD8^+^ T cells by reprogramming their metabolism in a GPR43-dependent manner during IAV infection (15). Specifically, *Blautia coccoides*-generated acetate reaches virus-specific CD8^+^ T cells *via* circulation, enters cells through MCTs (180), and is converted to acetyl-CoA by ACSS2 (**Figure 4**) (181). This acetyl-CoA replenishes the tricarboxylic acid (TCA) cycle pool, which is central to sugar, fat, and amino acid metabolism and energy production (182). The TCA cycle supplies ATP and electrons for antiviral gene transcription and protein synthesis, accelerating material cycling and transport (182, 183). Additionally, acetate treatment upregulates glucose transporter 1 (Glut-1) expression, enhancing glucose uptake for glycolysis (184). These metabolic changes are reflected in elevated oxygen consumption rate (OCR), mitochondrial mass, and extracellular acidification rate (ECAR) in virus-specific CD8^+^ T cells upon acetate exposure (15).

However, acetate-mediated metabolic reprogramming is contingent on GPR43 expression. Fueled by OXPHOS and glycolysis, GPR43 activation promotes IFN-*γ* and granzyme B production. IFN-*γ* activates macrophages *via* the JAK-STAT pathway to regulate T cell function (185), while granzyme B induces apoptosis in virus-infected cells through proteolysis and caspase activation (186). Collectively, acetate enhances intracellular acetyl-CoA synthesis and energy supply, optimizing virus-specific CD8^+^ T cell responses (15). The precise mechanisms by which acetate-GPR43 signaling modulates metabolism and effector molecule secretion remain unclear.

Butyrate, another SCFA, promotes cellular metabolism and memory potential in activated memory CD8^+^ T cells (125). Unlike acetate, butyrate uncouples glycolysis from the TCA cycle despite increasing glycolytic flux. Butyrate enters cells directly and is catabolized by activated CD8^+^ T cells, converted to acetyl-CoA *via β*-oxidation, and integrated into the TCA cycle (**Figure 4**) (187). This replacement of glycolysis-derived acetyl-CoA enables sustained OXPHOS through glutamine utilization and fatty acid catabolism, supporting long-term cell survival (125). Notably, butyrate-mediated metabolic reprogramming occurs independently of GPR41/43, whereas its role in enhancing memory CD8^+^ T cell differentiation during herpes simplex virus type 1 (HSV-1) infection is GPR41/43-dependent (125). Collectively, these findings demonstrate that butyrate not only optimizes CD8^+^ T cell metabolism to enhance antiviral effector functions but also coordinates innate and adaptive immune responses to resolve influenza infection. Specifically, butyrate accumulates alternatively activated macrophages and amplifies influenza-specific CD8^+^ T cell activity in lungs in a GPR41-dependent manner (20), thereby potentiating systemic antiviral immunity, alleviating host pathological damage, and enhancing rapid viral clearance. These multifaceted roles highlight SCFAs as critical mediators that bridge metabolic adaptation with immune defense against viral infections.

Qiu et al. did not explicitly distinguish whether acetate-mediated energy supply depends on GPR43 activation, whereas virus-specific CD8^+^ T cell differentiation induced by acetate is GPR43-dependent, consistent with findings by Bachem et al. (15, 125). This suggests that activation of the SCFA-GPCR-MAPK pathway plays a critical role in T cell differentiation, though further evidence is needed to characterize the mechanistic link between this pathway and cellular metabolism.

Regrettably, these findings had not yet furnished a comprehensive investigation into the specific contributions of HDACs in cellular differentiation, viral recognition, and metabolic reprogramming. This limitation may stem from the scope of the research articles and the priorities and perspectives of the investigators, which may preclude an exhaustive elucidation of each potential mechanism. Such constraints are indeed comprehensible. Nonetheless, it is imperative to consider these overarching mechanisms to attain a more profound comprehension of the operational dynamics of SCFAs.

## 6 Perspectives and limitations in understanding the gut microecosystem and SCFAs’ clinical applications

The gut microbiota, a dynamic ecosystem shaped by dietary, environmental, and host factors, plays a pivotal role in host health and antiviral immunity. Among its functional components, SCFAs emerge as central mediators of microbiota-host interactions, exerting pleiotropic effects on immune regulation, barrier integrity, and pathogen defense.

SCFAs, primarily produced by SCFA-producing bacteria, modulate immune responses through multiple mechanisms. By activating GPR43 signaling, they promote Treg expansion and B cell IgA production, fostering a balanced immune microenvironment that suppresses excessive inflammation and constrains microbial overgrowth (91, 188). Furthermore, butyrate and propionate inhibit DC development *via* HDAC-mediated repression of pro-inflammatory transcription factors (*e.g.*, PU.1, RelB), thereby reducing intestinal tissue inflammation (189). These effects collectively enhance mucosal integrity and antiviral defense, highlighting SCFAs as keystone metabolites in host-microbiota symbiosis.

Disruptions to SCFA-producing bacteria—such as those induced by antibiotics—severely compromise these protective functions. Reduced SCFA availability impairs Treg and IgA-mediated defenses (91, 188), increases intestinal epithelial permeability, and elevates susceptibility to viral infections. This underscores the urgent need for microbiota-targeted interventions, including dietary diversification (190, 191), controlled environmental microbial exposure (192, 193), and strategies to restore SCFA-producing communities after antibiotic use, to mitigate infection risk and preserve gut homeostasis.

Given the proximal colon as the primary site for endogenous SCFA absorption (194), optimizing SCFA delivery to this region is critical for maximizing their functional efficacy. As reviewed elsewhere (195), direct administration *via* rectal enemas or oral tablets offers viable strategies. Among formulation approaches, perfusion, microencapsulation, and enteric-coating significantly enhance blood-SCFA concentration, with the latter two demonstrating superior delivery efficiency and thus preferred clinical applicability (196–198). Alternative vehicles, such as high-amylose maize starch and acetylated/butyrylated starch, have also shown promise in mouse models (199, 200).

Dietary interventions provide a non-invasive route to elevate SCFA levels: fiber-rich diets and prebiotics (*e.g.*, inulin, guar gum) enrich SCFA-producing bacterial communities (201–203). However, their efficacy varies with fiber type, structural properties, dosage, and host-specific factors, including gut microbiota composition (204). Probiotic supplementation with live SCFA-producing bacteria presents another viable option (205), while fecal microbiota transplantation (FMT) from healthy donors offers a direct yet risk-laden approach (206, 207). The unpredictability of FMT outcomes—particularly the potential introduction of opportunistic pathogens in immunocompromised individuals—necessitates caution.

Current knowledge gaps underscore the need for further research on SCFA delivery systems, particularly regarding dosage optimization, recipient acceptance, and therapeutic efficacy in viral diseases. Advances in genetic engineering hold potential for designing synthetic symbiotic bacteria with enhanced SCFA synthesis capacity (208), though deciphering key SCFA synthesis genes remains a prerequisite.

Collectively, these strategies—ranging from advanced delivery systems to dietary interventions and microbial therapeutics—laid a foundation for personalized SCFA-based medicine. By tailoring interventions to individual microbiome profiles, such approaches could revolutionize the management of infections and chronic diseases.

Beyond their roles in antiviral immunity, SCFAs have been implicated in metabolic disorders (obesity, diabetes), systemic inflammation, and cancer (84, 157, 209–215). Yet, critical gaps remain in understanding their systemic mechanisms. For instance, while SCFAs modulate epigenetic landscapes and immune cell responsiveness, their evolutionary rationale—whether co-opted from bacterial survival strategies or intentionally optimized for host benefit—remains unresolved (216, 217). Addressing these questions is essential for translating SCFA-based therapies into clinical applications.

Future research should prioritize: (1) elucidating the multi-system effects of SCFAs through integrated omics approaches; (2) developing microbiota-based therapies to restore SCFA-producing communities in dysbiotic states (*e.g.*, probiotics, prebiotics, fecal transplantation); and (3) investigating the establishment and maintenance of SCFA-mediated symbiosis in early life, particularly during neonatal microbial colonization (218). These efforts will advance our understanding of host-microbiota co-evolution and pave the way for novel therapeutics against infectious and chronic diseases.

## 7 Conclusion

The health benefits of SCFAs and their microbial producers have been extensively documented, encompassing essential physiological functions such as digestion, immunity, and neurology. In the realm of immunity, the anti-inflammatory potential of SCFAs and their microbial producers has been well-characterized, with current research achieving significant progress in elucidating the detailed mechanisms underlying their role in antiviral immunity. While all SCFAs share a common role in anti-inflammatory processes, each SCFA can selectively and cooperatively activate unique pathways to combat viral invasion in specific cell types through HDAC-mediated, GPCR-mediated, and metabolic mechanisms. However, SCFAs exhibit context-dependent mechanisms with their immunomodulatory effects shaped by cellular metabolic and signaling states. The precise mechanisms governing the allocation and action of SCFAs within cells remain enigmatic. Future research should focus on deciphering these intracellular allocation mechanisms and highlight the importance of developing maintenance or reseeding protocols for the core microbiota of SCFA-producing bacteria in patients. Such efforts are highly meaningful for mitigating the adverse outcomes associated with viral infections.

## References

[1] Gordon JI. Honor thy gut symbionts redux. Science. (2012) 336:1251–3. doi: 10.1126/science.1224686, PMID: 22674326

[2] Levy M Blacher E Elinav E. Microbiome, metabolites and host immunity. Curr Opin Microbiol. (2017) 35:8–15. doi: 10.1016/j.mib.2016.10.003, PMID: 27883933

[3] Clemente JC Manasson J Scher JU. The role of the gut microbiome in systemic inflammatory disease. BMJ. (2018) 360:j5145. doi: 10.1136/bmj.j5145, PMID: 29311119 PMC6889978

[4] Guo W Zhou X Li X Zhu Q Peng J Zhu B. Depletion of gut microbiota impairs gut barrier function and antiviral immune defense in the liver. Front Immunol. (2021) 12:636803. doi: 10.3389/fimmu.2021.636803, PMID: 33841420 PMC8027085

[5] Gopalakrishnan V Spencer CN Nezi L Reuben A Andrews MC Karpinets TV. Gut microbiome modulates response to anti-PD-1 immunotherapy in melanoma patients. Science. (2018) 359:97–103. doi: 10.1126/science.aan4236, PMID: 29097493 PMC5827966

[6] Matson V Fessler J Bao R Chongsuwat T Zha Y Alegre ML. The commensal microbiome is associated with anti-PD-1 efficacy in metastatic melanoma patients. Science. (2018) 359:104–8. doi: 10.1126/science.aao3290, PMID: 29302014 PMC6707353

[7] Routy B Le Chatelier E Derosa L Duong CPM Alou MT Daillère R. Gut microbiome influences efficacy of PD-1-based immunotherapy against epithelial tumors. Science. (2018) 359:91–7. doi: 10.1126/science.aan3706, PMID: 29097494

[8] Derosa L Routy B Thomas AM Iebba V Zalcman G Friard S. Intestinal *Akkermansia muciniphila* predicts clinical response to PD-1 blockade in patients with advanced non-small-cell lung cancer. Nat Med. (2022) 28:315–24. doi: 10.1038/s41591-021-01655-5, PMID: 35115705 PMC9330544

[9] Effendi R Anshory M Kalim H Dwiyana RF Suwarsa O Pardo LM. *Akkermansia muciniphila* and *Faecalibacterium prausnitzii* in immune-related diseases. Microorganisms. (2022) 10:2382. doi: 10.3390/microorganisms10122382, PMID: 36557635 PMC9782003

[10] Li L Li M Chen Y Yu Z Cheng P Yu Z. Function and therapeutic prospects of next-generation probiotic *Akkermansia muciniphila* in infectious diseases. Front Microbiol. (2024) 15:1354447. doi: 10.3389/fmicb.2024.1354447, PMID: 38384263 PMC10880487

[11] VanderWaal K Deen J. Global trends in infectious diseases of swine. Proc Natl Acad Sci U.S.A. (2018) 115:11495–500. doi: 10.1073/pnas.1806068115, PMID: 30348781 PMC6233110

[12] Campbell DE Li Y Ingle H Baldridge MT. Impact of the microbiota on viral infections. Annu Rev Virol. (2023) 10:371–95. doi: 10.1146/annurev-virology-111821-115754, PMID: 37071931 PMC10543481

[13] Chakraborty C Sharma AR Bhattacharya M Dhama K Lee SS. Altered gut microbiota patterns in COVID-19: Markers for inflammation and disease severity. World J Gastroenterol. (2022) 28:2802–22. doi: 10.3748/wjg.v28.i25.2802, PMID: 35978881 PMC9280735

[14] Abd El-Hack ME El-Saadony MT Alqhtani AH Swelum AA Salem HM Elbestawy AR. The relationship among avian influenza, gut microbiota and chicken immunity: an updated overview. Poult Sci. (2022) 101:102021. doi: 10.1016/j.psj.2022.102021, PMID: 35939896 PMC9386105

[15] Qiu J Shi C Zhang Y Niu T Chen S Yang G. Microbiota-derived acetate is associated with functionally optimal virus-specific CD8^+^ T cell responses to influenza virus infection *via* GPR43-dependent metabolic reprogramming. Gut Microbes. (2024) 16:2401649. doi: 10.1080/19490976.2024.2401649, PMID: 39388633 PMC11469431

[16] Hays KE Pfaffinger JM Ryznar R. The interplay between gut microbiota, short-chain fatty acids, and implications for host health and disease. Gut Microbes. (2024) 16:2393270. doi: 10.1080/19490976.2024.2393270, PMID: 39284033 PMC11407412

[17] Chambers ES Preston T Frost G Morrison DJ. Role of gut microbiota-generated short-chain fatty acids in metabolic and cardiovascular health. Curr Nutr Rep. (2018) 7:198–206. doi: 10.1007/s13668-018-0248-8, PMID: 30264354 PMC6244749

[18] Silva YP Bernardi A Frozza RL. The role of short-chain fatty acids from gut microbiota in gut-brain communication. Front Endocrinol (Lausanne). (2020) 11:25. doi: 10.3389/fendo.2020.00025, PMID: 32082260 PMC7005631

[19] Liu P Wang Y Yang G Zhang Q Meng L Xin Y. The role of short-chain fatty acids in intestinal barrier function, inflammation, oxidative stress, and colonic carcinogenesis. Pharmacol Res. (2021) 165:105420. doi: 10.1016/j.phrs.2021.105420, PMID: 33434620

[20] Trompette A Gollwitzer ES Pattaroni C Lopez-Mejia IC Riva E Pernot J. Dietary fiber confers protection against flu by shaping Ly6c^-^ patrolling monocyte hematopoiesis and CD8^+^ T cell metabolism. Immunity. (2018) 48:992–1005.e8. doi: 10.1016/j.immuni.2018.04.022, PMID: 29768180

[21] Sun M-J Xing JH Yan Q-S Zou B-S Wang Y-J Niu T-M. The acetic acid produced by Lactobacillus species regulates immune function to alleviate PEDV infection in piglets. Probiotics Antimicro Prot. (2024). doi: 10.1007/s12602-024-10243-1, PMID: 38536635

[22] Antunes KH Fachi JL de Paula R da Silva EF Pral LP Dos Santos A. Microbiota-derived acetate protects against respiratory syncytial virus infection through a GPR43-type 1 interferon response. Nat Commun. (2019) 10:3273. doi: 10.1038/s41467-019-11152-6, PMID: 31332169 PMC6646332

[23] Antunes KH Stein RT FrancesChina C da Silva EF de Freitas DN Silveira J. Short-chain fatty acid acetate triggers antiviral response mediated by RIG-I in cells from infants with respiratory syncytial virus bronchiolitis. eBioMedicine. (2022) 77:103891. doi: 10.1016/j.ebiom.2022.103891, PMID: 35220042 PMC8871125

[24] Saint-Martin V Guillory V Chollot M Fleurot I Kut E Roesch F. The gut microbiota and its metabolite butyrate shape metabolism and antiviral immunity along the gut-lung axis in the chicken. Commun Biol. (2024) 7:1185. doi: 10.1038/s42003-024-06815-0, PMID: 39300162 PMC11413219

[25] Macfarlane S Macfarlane GT. Regulation of short-chain fatty acid production. Proc Nutr Soc. (2003) 62:67–72. doi: 10.1079/pns2002207, PMID: 12740060

[26] Liu XF Shao JH Liao YT Wang LN Jia Y Dong PJ. Regulation of short-chain fatty acids in the immune system. Front Immunol. (2023) 14:1186892. doi: 10.3389/fimmu.2023.1186892, PMID: 37215145 PMC10196242

[27] Cummings JH Pomare EW Branch WJ Naylor CP Macfarlane GT. Short chain fatty acids in human large intestine, portal, hepatic and venous blood. Gut. (1987) 28:1221–7. doi: 10.1136/gut.28.10.1221, PMID: 3678950 PMC1433442

[28] Topping DL Clifton PM. Short-chain fatty acids and human colonic function: roles of resistant starch and nonstarch polysaccharides. Physiol Rev. (2001) 81:1031–64. doi: 10.1152/physrev.2001.81.3.1031, PMID: 11427691

[29] Bloemen JG Venema K van de Poll MC Olde Damink SW Buurman WA Dejong CH. Short chain fatty acids exchange across the gut and liver in humans measured at surgery. Clin Nutr. (2009) 28:657–61. doi: 10.1016/j.clnu.2009.05.011, PMID: 19523724

[30] Xiong RG Zhou DD Wu SX Huang SY Saimaiti A Yang ZJ. Health benefits and side effects of short-chain fatty acids. Foods. (2022) 11:2863. doi: 10.3390/foods11182863, PMID: 36140990 PMC9498509

[31] Clausen MR Mortensen PB. Kinetic studies on colonocyte metabolism of short chain fatty acids and glucose in ulcerative colitis. Gut. (1995) 37:684–9. doi: 10.1136/gut.37.5.684, PMID: 8549946 PMC1382875

[32] Murase M Kimura Y Nagata Y. Determination of portal short-chain fatty acids in rats fed various dietary fibers by capillary gas chromatography. J Chromatogr B BioMed Appl. (1995) 664:415–20. doi: 10.1016/0378-4347(94)00491-m, PMID: 7780595

[33] Canfora EE Jocken JW Blaak EE. Short-chain fatty acids in control of body weight and insulin sensitivity. Nat Rev Endocrinol. (2015) 11:577–91. doi: 10.1038/nrendo.2015.128, PMID: 26260141

[34] Kim M Qie Y Park J Kim CH. Gut microbial metabolites fuel host antibody responses. Cell Host Microbe. (2016) 20:202–14. doi: 10.1016/j.chom.2016.07.001, PMID: 27476413 PMC4982788

[35] Miyauchi S Gopal E Fei Y-J Ganapathy V. Functional identification of SLC5A8, a tumor suppressor down-regulated in colon cancer, as a Na^+^-coupled transporter for short-chain fatty acids. J Biol Chem. (2004) 279:13293–6. doi: 10.1074/jbc.C400059200, PMID: 14966140

[36] Martin PM Dun Y Mysona B Ananth S Roon P Smith SB. Expression of the sodium-coupled monocarboxylate transporters SMCT1 (SLC5A8) and SMCT2 (SLC5A12) in retina. Invest Ophthalmol Vis Sci. (2007) 48:3356–63. doi: 10.1167/iovs.06-0888, PMID: 17591909

[37] Sepponen K Ruusunen M Pakkanen JA Pösö AR. Expression of CD147 and monocarboxylate transporters MCT1, MCT2 and MCT4 in porcine small intestine and colon. Vet J. (2007) 174:122–8. doi: 10.1016/j.tvjl.2006.05.015, PMID: 16901736

[38] Gurav A Sivaprakasam S Bhutia Yangzom D Boettger T Singh N Ganapathy V. Slc5a8, a Na^+^-coupled high-affinity transporter for short-chain fatty acids, is a conditional tumour suppressor in colon that protects against colitis and colon cancer under low-fibre dietary conditions. Biochem J. (2015) 469:267–78. doi: 10.1042/bj20150242, PMID: 25984582 PMC4943859

[39] Halestrap AP Meredith D. The SLC16 gene family—from monocarboxylate transporters (MCTs) to aromatic amino acid transporters and beyond. Pflugers Arch. (2004) 447:619–28. doi: 10.1007/s00424-003-1067-2, PMID: 12739169

[40] den Besten G van Eunen K Groen AK Venema K Reijngoud D-J Bakker BM. The role of short-chain fatty acids in the interplay between diet, gut microbiota, and host energy metabolism. J Lipid Res. (2013) 54:2325–40. doi: 10.1194/jlr.R036012, PMID: 23821742 PMC3735932

[41] Roth SY Denu JM Allis CD. Histone acetyltransferases. Annu Rev Biochem. (2001) 70:81–120. doi: 10.1146/annurev.biochem.70.1.81, PMID: 11395403

[42] Fredriksson R Lagerström MC Lundin L-G Schiöth HB. The G-protein-coupled receptors in the human genome form five main families. phylogenetic analysis, paralogon groups, and fingerprints. Mol Pharmacol. (2003) 63:1256–72. doi: 10.1124/mol.63.6.1256, PMID: 12761335

[43] Brown AJ Goldsworthy SM Barnes AA Eilert MM Tcheang L Daniels D. The orphan G protein-coupled receptors GPR41 and GPR43 are activated by propionate and other short chain carboxylic acids. J Biol Chem. (2003) 278:11312–9. doi: 10.1074/jbc.M211609200, PMID: 12496283

[44] Singh N Gurav A Sivaprakasam S Brady E Padia R Shi H. Activation of Gpr109a, receptor for niacin and the commensal metabolite butyrate, suppresses colonic inflammation and carcinogenesis. Immunity. (2014) 40:128–39. doi: 10.1016/j.immuni.2013.12.007, PMID: 24412617 PMC4305274

[45] Le Poul E Loison C Struyf S Springael J-Y Lannoy V Decobecq M-E. Functional characterization of human receptors for short chain fatty acids and their role in polymorphonuclear cell activation. J Biol Chem. (2003) 278:25481–9. doi: 10.1074/jbc.M301403200, PMID: 12711604

[46] Thangaraju M Cresci GA Liu K Ananth S Gnanaprakasam JP Browning DD. GPR109A is a G-protein-coupled receptor for the bacterial fermentation product butyrate and functions as a tumor suppressor in colon. Cancer Res. (2009) 69:2826–32. doi: 10.1158/0008-5472.CAN-08-4466, PMID: 19276343 PMC3747834

[47] van der Hee B Wells JM. Microbial regulation of host physiology by short-chain fatty acids. Trends Microbiol. (2021) 29:700–12. doi: 10.1016/j.tim.2021.02.001, PMID: 33674141

[48] Oldham WM Hamm HE. Heterotrimeric G protein activation by G-protein-coupled receptors. Nat Rev Mol Cell Biol. (2008) 9:60–71. doi: 10.1038/nrm2299, PMID: 18043707

[49] Wilkie TM Gilbert DJ Olsen AS Chen XN Amatruda TT Korenberg JR. Evolution of the mammalian G protein *α* subunit multigene family. Nat Genet. (1992) 1:85–91. doi: 10.1038/ng0592-85, PMID: 1302014

[50] Rosenbaum DM Rasmussen SGF Kobilka BK. The structure and function of G-protein-coupled receptors. Nature. (2009) 459:356–63. doi: 10.1038/nature08144, PMID: 19458711 PMC3967846

[51] Melhem H Kaya B Ayata CK Hruz P Niess JH. Metabolite-sensing G protein-coupled receptors connect the diet-microbiota-metabolites axis to inflammatory bowel disease. Cells. (2019) 8(5):450. doi: 10.3390/cells8050450, PMID: 31091682 PMC6562883

[52] Kristiansen K. Molecular mechanisms of ligand binding, signaling, and regulation within the superfamily of G-protein-coupled receptors: molecular modeling and mutagenesis approaches to receptor structure and function. Pharmacol Ther. (2004) 103:21–80. doi: 10.1016/j.pharmthera.2004.05.002, PMID: 15251227

[53] Freedman NJ Lefkowitz RJ. Desensitization of G protein-coupled receptors. Recent Prog Horm Res. (1996) 51:319–51. 8701085

[54] Grimes J Koszegi Z Lanoiselée Y Miljus T O’Brien SL Stepniewski TM. Plasma membrane preassociation drives *β*-arrestin coupling to receptors and activation. Cell. (2023) 186:2238–55.e20. doi: 10.1016/j.cell.2023.04.018, PMID: 37146613 PMC7614532

[55] Pierce KL Lefkowitz RJ. Classical and new roles of *β*-arrestins in the regulation of G-protein-coupled receptors. Nat Rev Neurosci. (2001) 2:727–33. doi: 10.1038/35094577, PMID: 11584310

[56] Pöll F Doll C Schulz S. Rapid dephosphorylation of G protein-coupled receptors by protein phosphatase 1*β* is required for termination of *β*-arrestin-dependent signaling. J Biol Chem. (2011) 286:32931–6. doi: 10.1074/jbc.M111.224899, PMID: 21795688 PMC3190940

[57] Yu SS Lefkowitz RJ Hausdorff WP. Beta-adrenergic receptor sequestration. A potential mechanism of receptor resensitization. J Biol Chem. (1993) 268:337–41. doi: 10.1016/S0021-9258(18)54155-7, PMID: 8380158

[58] Lohse MJ Benovic JL Codina J Caron MG Lefkowitz RJ. *β*-Arrestin: a protein that regulates *β*-adrenergic receptor function. Science. (1990) 248:1547–50. doi: 10.1126/science.2163110, PMID: 2163110

[59] Luttrell LM Ferguson SSG Daaka Y Miller WE Maudsley S Della Rocca GJ. *β*-Arrestin-dependent formation of *β*2 adrenergic receptor-Src protein kinase complexes. Science. (1999) 283:655–61. doi: 10.1126/science.283.5402.655, PMID: 9924018

[60] McDonald PH Chow C-W Miller WE Laporte SA Field ME Lin F-T. *β*-Arrestin 2: a receptor-regulated MAPK scaffold for the activation of JNK3. Science. (2000) 290:1574–7. doi: 10.1126/science.290.5496.1574, PMID: 11090355

[61] DeFea KA Vaughn ZD O’Bryan EM Nishijima D Déry O Bunnett NW. The proliferative and antiapoptotic effects of substance P are facilitated by formation of a *β*-arrestin-dependent scaffolding complex. Proc Natl Acad Sci USA. (2000) 97:11086–91. doi: 10.1073/pnas.190276697, PMID: 10995467 PMC27152

[62] Azzi M Charest PG Angers S Rousseau G Kohout T Bouvier M. *β*-Arrestin-mediated activation of MAPK by inverse agonists reveals distinct active conformations for G protein-coupled receptors. Proc Natl Acad Sci USA. (2003) 100:11406–11. doi: 10.1073/pnas.1936664100, PMID: 13679574 PMC208770

[63] Houslay MD Milligan G. Tailoring cAMP-signalling responses through isoform multiplicity. Trends Biochem Sci. (1997) 22:217–24. doi: 10.1016/S0968-0004(97)01050-5, PMID: 9204709

[64] Mizuta K Sasaki H Zhang Y Matoba A Emala CW. The short-chain free fatty acid receptor FFAR3 is expressed and potentiates contraction in human airway smooth muscle. Am J Physiol Lung Cell Mol Physiol. (2020) 318:L1248–L60. doi: 10.1152/ajplung.00357.2019, PMID: 32209026 PMC7347267

[65] Priyadarshini M Layden BT. FFAR3 modulates insulin secretion and global gene expression in mouse islets. Islets. (2015) 7:e1045182. doi: 10.1080/19382014.2015.1045182, PMID: 26091414 PMC4878265

[66] Hamm HE. The many faces of G protein signaling. J Biol Chem. (1998) 273:669–72. doi: 10.1074/jbc.273.2.669, PMID: 9422713

[67] Lei Q Jones MB Talley EM Garrison JC Bayliss DA. Molecular mechanisms mediating inhibition of G protein-coupled inwardly-rectifying K^+^ channels. Mol Cells. (2003) 15:1–9. doi: 10.1016/S1016-8478(23)13700-9 12661754

[68] Kimura I Inoue D Maeda T Hara T Ichimura A Miyauchi S. Short-chain fatty acids and ketones directly regulate sympathetic nervous system *via* G protein-coupled receptor 41 (GPR41). Proc Natl Acad Sci USA. (2011) 108:8030–5. doi: 10.1073/pnas.1016088108, PMID: 21518883 PMC3093469

[69] Rhee SG Bae YS. Regulation of phosphoinositide-specific phospholipase C isozymes. J Biol Chem. (1997) 272:15045–8. doi: 10.1074/jbc.272.24.15045, PMID: 9182519

[70] Berridge MJ Irvine RF. Inositol trisphosphate, a novel second messenger in cellular signal transduction. Nature. (1984) 312:315–21. doi: 10.1038/312315a0, PMID: 6095092

[71] Berridge MJ. Inositol trisphosphate and calcium signalling. Nature. (1993) 361:315–25. doi: 10.1038/361315a0, PMID: 8381210

[72] Nishizuka Y. The role of protein kinase C in cell surface signal transduction and tumour promotion. Nature. (1984) 308:693–8. doi: 10.1038/308693a0, PMID: 6232463

[73] Nishizuka Y. The molecular heterogeneity of protein kinase C and its implications for cellular regulation. Nature. (1988) 334:661–5. doi: 10.1038/334661a0, PMID: 3045562

[74] Gutkind JS. Regulation of mitogen-activated protein kinase signaling networks by G protein-coupled receptors. Sci STKE. (2000) 2000:re1. doi: 10.1126/stke.2000.40.re1, PMID: 11752597

[75] Dekker LV Segal AW. Signals to move cells. Science. (2000) 287:982–5. doi: 10.1126/science.287.5455.982, PMID: 10691572

[76] Katso R Okkenhaug K Ahmadi K White S Timms J Waterfield MD. Cellular function of phosphoinositide 3-kinases: implications for development, homeostasis, and cancer. Annu Rev Cell Dev Biol. (2001) 17:615–75. doi: 10.1146/annurev.cellbio.17.1.615, PMID: 11687500

[77] Downward J. Control of ras activation. Cancer Surv. (1996) 27:87–100. 8909796

[78] Lopez-Ilasaca M Crespo P Pellici PG Gutkind JS Wetzker R. Linkage of G protein-coupled receptors to the MAPK signaling pathway through PI 3-kinase gamma. Science. (1997) 275:394–7. doi: 10.1126/science.275.5298.394, PMID: 8994038

[79] Shajahan AN Tiruppathi C Smrcka AV Malik AB Minshall RD. G*βγ* activation of Src induces caveolae-mediated endocytosis in endothelial cells. J Biol Chem. (2004) 279:48055–62. doi: 10.1074/jbc.M405837200, PMID: 15345719

[80] Chessa TAM Jung P Anwar A Suire S Anderson KE Barneda D. PLEKHS1 drives PI3Ks and remodels pathway homeostasis in PTEN-null prostate. Mol Cell. (2023) 83:2991–3009.e13. doi: 10.1016/j.molcel.2023.07.015, PMID: 37567175

[81] Liu L Puri KD Penninger JM Kubes P. Leukocyte PI3K*γ* and PI3K*δ* have temporally distinct roles for leukocyte recruitment in *vivo* . Blood. (2007) 110:1191–8. doi: 10.1182/blood-2006-11-060103, PMID: 17488877

[82] Li G Deng X Wu C Zhou Q Chen L Shi Y. Distinct kinetic and spatial patterns of protein kinase C (PKC)- and epidermal growth factor receptor (EGFR)-dependent activation of extracellular signal-regulated kinases 1 and 2 by human nicotinic acid receptor GPR109A. J Biol Chem. (2011) 286:31199–212. doi: 10.1074/jbc.M111.241372, PMID: 21768093 PMC3173092

[83] Bauset C Carda-Diéguez M Cejudo-Garcés A Buetas E Seco-Cervera M Macias-Ceja DC. A disturbed metabolite-GPCR axis is associated with microbial dysbiosis in IBD patients: Potential role of GPR109A in macrophages. Biochim Biophys Acta. (2024) 1870:167489. doi: 10.1016/j.bbadis.2024.167489, PMID: 39233260

[84] Vinolo MA Rodrigues HG Nachbar RT Curi R. Regulation of inflammation by short chain fatty acids. Nutrients. (2011) 3:858–76. doi: 10.3390/nu3100858, PMID: 22254083 PMC3257741

[85] Song W Li D Tao L Luo Q Chen L. Solute carrier transporters: the metabolic gatekeepers of immune cells. Acta Pharm Sin B. (2020) 10:61–78. doi: 10.1016/j.apsb.2019.12.006, PMID: 31993307 PMC6977534

[86] Kim EK Choi E-J. Pathological roles of MAPK signaling pathways in human diseases. Biochim Biophys Acta. (2010) 1802:396–405. doi: 10.1016/j.bbadis.2009.12.009, PMID: 20079433

[87] Salvador-Bernáldez M Mateus SB Del Barco Barrantes I Arthur SC Martínez AC Nebreda AR. p38*α* regulates cytokine-induced IFN*γ* secretion *via* the Mnk1/eIF4E pathway in Th1 cells. Immunol Cell Biol. (2017) 95:814–23. doi: 10.1038/icb.2017.51, PMID: 28611474

[88] Trompette A Gollwitzer ES Yadava K Sichelstiel AK Sprenger N Ngom-Bru C. Gut microbiota metabolism of dietary fiber influences allergic airway disease and hematopoiesis. Nat Med. (2014) 20:159–66. doi: 10.1038/nm.3444, PMID: 24390308

[89] Zhong S Li H Wang YS Wang Y Ji G Li HY. Bmp8a is an essential positive regulator of antiviral immunity in zebrafish. Commun Biol. (2021) 4:318. doi: 10.1038/s42003-021-01811-0, PMID: 33750893 PMC7943762

[90] Alaoui L Palomino G Zurawski S Zurawski G Coindre S Dereuddre-Bosquet N. Early SIV and HIV infection promotes the LILRB2/MHC-I inhibitory axis in cDCs. Cell Mol Life Sci. (2018) 75:1871–87. doi: 10.1007/s00018-017-2712-9, PMID: 29134249 PMC11105587

[91] Smith PM Howitt MR Panikov N Michaud M Gallini CA Bohlooly-Y M. The microbial metabolites, short-chain fatty acids, regulate colonic T_reg_ cell homeostasi. Science. (2013) 341:569–73. doi: 10.1126/science.1241165, PMID: 23828891 PMC3807819

[92] Kim MH Kang SG Park JH Yanagisawa M Kim CH. Short-chain fatty acids activate GPR41 and GPR43 on intestinal epithelial cells to promote inflammatory responses in mice. Gastroenterology. (2013) 145:396–406.e1-10. doi: 10.1053/j.gastro.2013.04.056, PMID: 23665276

[93] Kimura I Ichimura A Ohue-Kitano R Igarashi M. Free fatty acid receptors in health and disease. Physiol Rev. (2019) 100:171–210. doi: 10.1152/physrev.00041.2018, PMID: 31487233

[94] Satagopam VP Theodoropoulou MC Stampolakis CK Pavlopoulos GA Papandreou NC Bagos PG. GPCRs, G-proteins, effectors and their interactions: human-gpDB, a database employing visualization tools and data integration techniques. Database (Oxford). (2010) 2010:baq019. doi: 10.1093/database/baq019, PMID: 20689020 PMC2931634

[95] Masuho I Ostrovskaya O Kramer GM Jones CD Xie K Martemyanov KA. Distinct profiles of functional discrimination among G proteins determine the actions of G protein-coupled receptors. Sci Signal. (2015) 8:ra123. doi: 10.1126/scisignal.aab4068, PMID: 26628681 PMC4886239

[96] Inoue A Raimondi F Kadji FMN Singh G Kishi T Uwamizu A. Illuminating G-protein-coupling selectivity of GPCRs. Cell. (2019) 177:1933–47.e25. doi: 10.1016/j.cell.2019.04.044, PMID: 31160049 PMC6773469

[97] Kapolka NJ Taghon GJ Rowe JB Morgan WM Enten JF Lambert NA. DCyFIR: a high-throughput CRISPR platform for multiplexed G protein-coupled receptor profiling and ligand discovery. Proc Natl Acad Sci USA. (2020) 117:13117–26. doi: 10.1073/pnas.2000430117, PMID: 32434907 PMC7293659

[98] Okashah N Wright SC Kawakami K Mathiasen S Zhou J Lu S. Agonist-induced formation of unproductive receptor-G_12_ complexes. Proc Natl Acad Sci USA. (2020) 117:21723–30. doi: 10.1073/pnas.2003787117, PMID: 32817560 PMC7474645

[99] Olsen RHJ DiBerto JF English JG Glaudin AM Krumm BE Slocum ST. TRUPATH, an open-source biosensor platform for interrogating the GPCR transducerome. Nat Chem Biol. (2020) 16:841–9. doi: 10.1038/s41589-020-0535-8, PMID: 32367019 PMC7648517

[100] Avet C Mancini A Breton B Le Gouill C Hauser AS Normand C. Effector membrane translocation biosensors reveal G protein and *β*arrestin coupling profiles of 100 therapeutically relevant GPCRs. eLife. (2022) 11:e74101. doi: 10.7554/eLife.74101, PMID: 35302493 PMC9005190

[101] Masuho I Kise R Gainza P Von Moo E Li X Tany R. Rules and mechanisms governing G protein coupling selectivity of GPCRs. Cell Rep. (2023) 42:113173. doi: 10.1016/j.celrep.2023.113173, PMID: 37742189 PMC10842385

[102] Klein Herenbrink C Sykes DA Donthamsetti P Canals M Coudrat T Shonberg J. The role of kinetic context in apparent biased agonism at GPCRs. Nat Commun. (2016) 7:10842. doi: 10.1038/ncomms10842, PMID: 26905976 PMC4770093

[103] Arpaia N Campbell C Fan X Dikiy S van der Veeken J deRoos P. Metabolites produced by commensal bacteria promote peripheral regulatory T-cell generation. Nature. (2013) 504:451–5. doi: 10.1038/nature12726, PMID: 24226773 PMC3869884

[104] Whitehead RH Young GP Bhathal PS. Effects of short chain fatty acids on a new human colon carcinoma cell line (LIM1215). Gut. (1986) 27:1457–63. doi: 10.1136/gut.27.12.1457, PMID: 3804021 PMC1433981

[105] Gamet L Daviaud D Denis-Pouxviel C Remesy C Murat JC. Effects of short-chain fatty acids on growth and differentiation of the human colon-cancer cell line HT29. Int J Cancer. (1992) 52:286–9. doi: 10.1002/ijc.2910520222, PMID: 1521915

[106] Basson MD Turowski GA Rashid Z Hong F Madri JA. Regulation of human colonic cell line proliferation and phenotype by sodium butyrate. Dig Dis Sci. (1996) 41:1989–93. doi: 10.1007/bf02093601, PMID: 8888712

[107] Siavoshian S Blottière HM Le Foll E Kaeffer B Cherbut C Galmiche JP. Comparison of the effect of different short chain fatty acids on the growth and differentiation of human colonic carcinoma cell lines *in vitro* . Cell Biol Int. (1997) 21:281–7. doi: 10.1006/cbir.1997.0153, PMID: 9243803

[108] Sakata T. Stimulatory effect of short-chain fatty acids on epithelial cell proliferation in the rat intestine: a possible explanation for trophic effects of fermentable fibre, gut microbes and luminal trophic factors. Br J Nutr. (1987) 58:95–103. doi: 10.1079/bjn19870073, PMID: 3620440

[109] Frankel WL Zhang W Singh A Klurfeld DM Don S Sakata T. Mediation of the trophic effects of short-chain fatty acids on the rat jejunum and colon. Gastroenterology. (1994) 106:375–80. doi: 10.1016/0016-5085(94)90595-9, PMID: 8299904

[110] Kien CL Blauwiekel R Bunn JY Jetton TL Frankel WL Holst JJ. Cecal infusion of butyrate increases intestinal cell proliferation in piglets. J Nutr. (2007) 137:916–22. doi: 10.1093/jn/137.4.916, PMID: 17374654 PMC1949321

[111] Comalada M Bailón E de Haro O Lara-Villoslada F Xaus J Zarzuelo A. The effects of short-chain fatty acids on colon epithelial proliferation and survival depend on the cellular phenotype. J Cancer Res Clin Oncol. (2006) 132:487–97. doi: 10.1007/s00432-006-0092-x, PMID: 16788843 PMC12161102

[112] Mariadason JM Velcich A Wilson AJ Augenlicht LH Gibson PR. Resistance to butyrate-induced cell differentiation and apoptosis during spontaneous Caco-2 cell differentiation. Gastroenterology. (2001) 120:889–99. doi: 10.1053/gast.2001.22472, PMID: 11231943

[113] Salvi PS Cowles RA. Butyrate and the intestinal epithelium: modulation of proliferation and inflammation in homeostasis and disease. Cells. (2021) 10:1775. doi: 10.3390/cells10071775, PMID: 34359944 PMC8304699

[114] Furusawa Y Obata Y Fukuda S Endo TA Nakato G Takahashi D. Commensal microbe-derived butyrate induces the differentiation of colonic regulatory T cells. Nature. (2013) 504:446–50. doi: 10.1038/nature12721, PMID: 24226770

[115] Warburg O. On the origin of cancer cells. Science. (1956) 123:309–14. doi: 10.1126/science.123.3191.309, PMID: 13298683

[116] Vander Heiden MG Cantley LC Thompson CB. Understanding the Warburg effect: the metabolic requirements of cell proliferation. Science. (2009) 324:1029–33. doi: 10.1126/science.1160809, PMID: 19460998 PMC2849637

[117] Blouin JM Penot G Collinet M Nacfer M Forest C Laurent-Puig P. Butyrate elicits a metabolic switch in human colon cancer cells by targeting the pyruvate dehydrogenase complex. Int J Cancer. (2011) 128:2591–601. doi: 10.1002/ijc.25599, PMID: 20715114

[118] Li Q Cao L Tian Y Zhang P Ding C Lu W. Butyrate suppresses the proliferation of colorectal cancer cells *via* targeting pyruvate kinase M2 and metabolic reprogramming. Mol Cell Proteomics. (2018) 17:1531–45. doi: 10.1074/mcp.RA118.000752, PMID: 29739823 PMC6072541

[119] Geng H-W Yin F-Y Zhang Z-F Gong X Yang Y. Butyrate suppresses glucose metabolism of colorectal cancer cells *via* GPR109a-AKT signaling pathway and enhances chemotherapy. Front Mol Biosci. (2021) 8:634874. doi: 10.3389/fmolb.2021.634874, PMID: 33855046 PMC8039130

[120] Sun H Chen L Cao S Liang Y Xu Y. Warburg effects in cancer and normal proliferating cells: two tales of the same name. Genomics Proteomics Bioinf. (2019) 17:273–86. doi: 10.1016/j.gpb.2018.12.006, PMID: 31071451 PMC6818181

[121] Combs JA Norton EB Saifudeen ZR Bentrup KHZ Katakam PV Morris CA. Human cytomegalovirus alters host cell mitochondrial function during acute infection. J Virol. (2020) 94:e01183–011819. doi: 10.1128/jvi.01183-19, PMID: 31694945 PMC6955246

[122] He Y Fu L Li Y Wang W Gong M Zhang J. Gut microbial metabolites facilitate anticancer therapy efficacy by modulating cytotoxic CD8^+^ T cell immunity. Cell Metab. (2021) 33:988–1000.e7. doi: 10.1016/j.cmet.2021.03.002, PMID: 33761313

[123] Donohoe DR Garge N Zhang X Sun W O’Connell TM Bunger MK. The microbiome and butyrate regulate energy metabolism and autophagy in the mammalian colon. Cell Metab. (2011) 13:517–26. doi: 10.1016/j.cmet.2011.02.018, PMID: 21531334 PMC3099420

[124] Kaiko GE Ryu SH Koues OI Collins PL Solnica-Krezel L Pearce EJ. The colonic crypt protects stem cells from microbiota-derived metabolites. Cell. (2016) 165:1708–20. doi: 10.1016/j.cell.2016.05.018, PMID: 27264604 PMC5026192

[125] Bachem A Makhlouf C Binger KJ de Souza DP Tull D Hochheiser K. Microbiota-derived short-chain fatty acids promote the memory potential of antigen-activated CD8^+^ T cells. Immunity. (2019) 51:285–97.e5. doi: 10.1016/j.immuni.2019.06.002, PMID: 31272808

[126] Peng K Xiao S Xia S Li C Yu H Yu Q. Butyrate inhibits the HDAC8/NF-*κ*B pathway to enhance Slc26a3 expression and improve the intestinal epithelial barrier to relieve colitis. J Agric Food Chem. (2024) 72:24400–16. doi: 10.1021/acs.jafc.4c04456, PMID: 39440960

[127] Sencio V Gallerand A MaChado MG Deruyter L Heumel S Soulard D. Influenza virus infection impairs the gut’s barrier properties and favors secondary enteric bacterial infection through reduced production of short-chain fatty acids. Infect Immun. (2021) 89:e0073420. doi: 10.1128/iai.00734-20, PMID: 33820816 PMC8370677

[128] Wang G Liu J Zhang Y Xie J Chen S Shi Y. Ginsenoside Rg3 enriches SCFA-producing commensal bacteria to confer protection against enteric viral infection via the cGAS-STING-type I IFN axis. ISME J. (2023) 17:2426–40. doi: 10.1038/s41396-023-01541-7, PMID: 37950067 PMC10689736

[129] Chemudupati M Smith AC Fillinger RJ Kenney AD Zhang L Zani A. Short chain fatty acid butyrate promotes virus infection by repressing interferon stimulated genes. bioRxiv. (2020). doi: 10.1101/2020.02.04.934919

[130] Yin L Liu X Yao Y Yuan M Luo Y Zhang G. Gut microbiota-derived butyrate promotes coronavirus TGEV infection through impairing RIG-I-triggered local type I interferon responses via class I HDAC inhibition. J Virol. (2024) 98:e01377–23. doi: 10.1128/jvi.01377-23, PMID: 38197629 PMC10878070

[131] Chen Y Sprung R Tang Y Ball H Sangras B Kim SC. Lysine propionylation and butyrylation are novel post-translational modifications in histones. Mol Cell Proteomics. (2007) 6:812–9. doi: 10.1074/mcp.M700021-MCP200, PMID: 17267393 PMC2911958

[132] Dai L Peng C Montellier E Lu Z Chen Y Ishii H. Lysine 2-hydroxyisobutyrylation is a widely distributed active histone mark. Nat Chem Biol. (2014) 10:365–70. doi: 10.1038/nchembio.1497, PMID: 24681537

[133] Fellows R Denizot J Stellato C Cuomo A Jain P Stoyanova E. Microbiota derived short chain fatty acids promote histone crotonylation in the colon through histone deacetylases. Nat Commun. (2018) 9:105. doi: 10.1038/s41467-017-02651-5, PMID: 29317660 PMC5760624

[134] Sabari BR Tang Z Huang H Yong-Gonzalez V Molina H Kong HE. Intracellular crotonyl-CoA stimulates transcription through p300-catalyzed histone crotonylation. Mol Cell. (2015) 58:203–15. doi: 10.1016/j.molcel.2015.02.029, PMID: 25818647 PMC4501262

[135] Sabari BR Zhang D Allis CD Zhao Y. Metabolic regulation of gene expression through histone acylations. Nat Rev Mol Cell Biol. (2017) 18:90–101. doi: 10.1038/nrm.2016.140, PMID: 27924077 PMC5320945

[136] Zeaiter N Belot L Cunin V Nahed RA Tokarska-Schlattner M Le Gouellec A. Acetyl-CoA synthetase (ACSS2) does not generate butyryl- and crotonyl-CoA. Mol Metab. (2024) 81:101903. doi: 10.1016/j.molmet.2024.101903, PMID: 38369012 PMC10906504

[137] Nagesh PT Husain M. Influenza A virus dysregulates host histone deacetylase 1 that inhibits viral infection in lung epithelial cells. J Virol. (2016) 90:4614–25. doi: 10.1128/jvi.00126-16, PMID: 26912629 PMC4836332

[138] Jiang G Nguyen D Archin NM Yukl SA Méndez-Lagares G Tang Y. HIV latency is reversed by ACSS2-driven histone crotonylation. J Clin Invest. (2018) 128:1190–8. doi: 10.1172/JCI98071, PMID: 29457784 PMC5824862

[139] Nshanian M Gruber JJ Geller BS Chleilat F Lancaster SM White SM. Short-chain fatty acid metabolites propionate and butyrate are unique epigenetic regulatory elements linking diet, metabolism and gene expression. Nat Metab. (2025) 7:196–211. doi: 10.1038/s42255-024-01191-9, PMID: 39789354 PMC11774759

[140] Montgomery DC Sorum AW Meier JL. Chemoproteomic profiling of lysine acetyltransferases highlights an expanded landscape of catalytic acetylation. J Am Chem Soc. (2014) 136:8669–76. doi: 10.1021/ja502372j, PMID: 24836640 PMC4227742

[141] Simithy J Sidoli S Yuan ZF Coradin M Bhanu NV Marchione DM. Characterization of histone acylations links chromatin modifications with metabolism. Nat Commun. (2017) 8:1141. doi: 10.1038/s41467-017-01384-9, PMID: 29070843 PMC5656686

[142] Brownell JE Zhou J Ranalli T Kobayashi R Edmondson DG Roth SY. Tetrahymena histone acetyltransferase A: a homolog to yeast Gcn5p linking histone acetylation to gene activation. Cell. (1996) 84:843–51. doi: 10.1016/s0092-8674(00)81063-6, PMID: 8601308

[143] Chen L Fischle W Verdin E Greene WC. Duration of nuclear NF-kappaB action regulated by reversible acetylation. Science. (2001) 293:1653–7. doi: 10.1126/science.1062374, PMID: 11533489

[144] Huang B Yang XD Lamb A Chen LF. Posttranslational modifications of NF-kappaB: another layer of regulation for NF-kappaB signaling pathway. Cell Signal. (2010) 22:1282–90. doi: 10.1016/j.cellsig.2010.03.017, PMID: 20363318 PMC2893268

[145] Kim M Lu F Zhang Y. Loss of HDAC-mediated repression and gain of NF-*κ*B activation underlie cytokine induction in ARID1A- and PIK3CA-mutation-driven ovarian cancer. Cell Rep. (2016) 17:275–88. doi: 10.1016/j.celrep.2016.09.003, PMID: 27681437 PMC7734570

[146] Almeida LO Abrahao AC Rosselli-Murai LK Giudice FS Zagni C Leopoldino AM. NF*κ*B mediates cisplatin resistance through histone modifications in head and neck squamous cell carcinoma (HNSCC). FEBS Open Bio. (2014) 4:96–104. doi: 10.1016/j.fob.2013.12.003, PMID: 24490130 PMC3907686

[147] Basagoudanavar SH Thapa RJ Nogusa S Wang J Beg AA Balachandran S. Distinct roles for the NF-kappa B RelA subunit during antiviral innate immune responses. J Virol. (2011) 85:2599–610. doi: 10.1128/jvi.02213-10, PMID: 21209118 PMC3067974

[148] Chen X Barozzi I Termanini A Prosperini E Recchiuti A Dalli J. Requirement for the histone deacetylase Hdac3 for the inflammatory gene expression program in macrophages. Proc Natl Acad Sci U.S.A. (2012) 109:E2865–74. doi: 10.1073/pnas.1121131109, PMID: 22802645 PMC3479529

[149] Zhu H Shan L Schiller PW Mai A Peng T. Histone deacetylase-3 activation promotes tumor necrosis factor-alpha (TNF-alpha) expression in cardiomyocytes during lipopolysaccharide stimulation. J Biol Chem. (2010) 285:9429–36. doi: 10.1074/jbc.M109.071274, PMID: 20097764 PMC2843192

[150] Leus NG Zwinderman MR Dekker FJ. Histone deacetylase 3 (HDAC 3) as emerging drug target in NF-*κ*B-mediated inflammation. Curr Opin Chem Biol. (2016) 33:160–8. doi: 10.1016/j.cbpa.2016.06.019, PMID: 27371876 PMC5019345

[151] Wang X Xia H Liu S Cao L You F. Epigenetic regulation in antiviral innate immunity. Eur J Immunol. (2021) 51:1641–51. doi: 10.1002/eji.202048975, PMID: 33964027

[152] Meng J Liu X Zhang P Li D Xu S Zhou Q. Rb selectively inhibits innate IFN-*β* production by enhancing deacetylation of IFN-*β* promoter through HDAC1 and HDAC8. J Autoimmun. (2016) 73:42–53. doi: 10.1016/j.jaut.2016.05.012, PMID: 27267461

[153] Chen L Wang C Luo J Su W Li M Zhao N. Histone deacetylase 1 plays an acetylation-independent role in influenza A virus replication. Front Immunol. (2017) 8:1757. doi: 10.3389/fimmu.2017.01757, PMID: 29312300 PMC5733105

[154] Williams SA Chen LF Kwon H Ruiz-Jarabo CM Verdin E Greene WC. NF-kappaB p50 promotes HIV latency through HDAC recruitment and repression of transcriptional initiation. EMBO J. (2006) 25:139–49. doi: 10.1038/sj.emboj.7600900, PMID: 16319923 PMC1356344

[155] Adam E Quivy V Bex F Chariot A Collette Y Vanhulle C. Potentiation of tumor necrosis factor-induced NF-kappa B activation by deacetylase inhibitors is associated with a delayed cytoplasmic reappearance of I kappa B alpha. Mol Cell Biol. (2003) 23:6200–9. doi: 10.1128/mcb.23.17.6200-6209.2003, PMID: 12917341 PMC180966

[156] Huang A Cai R Wang Q Shi L Li C Yan H. Dynamic change of gut microbiota during porcine epidemic diarrhea virus infection in suckling piglets. Front Microbiol. (2019) 10:322. doi: 10.3389/fmicb.2019.00322, PMID: 30858839 PMC6397872

[157] Morales-Ferré C Azagra-Boronat I Massot-Cladera M Tims S Knipping K Garssen J. Preventive effect of a postbiotic and prebiotic mixture in a rat model of early life rotavirus induced-diarrhea. Nutrients. (2022) 14:1163. doi: 10.3390/nu14061163, PMID: 35334820 PMC8954028

[158] Mauri C Gray D Mushtaq N Londei M. Prevention of arthritis by interleukin 10–producing B cells. J Exp Med. (2003) 197:489–501. doi: 10.1084/jem.20021293, PMID: 12591906 PMC2193864

[159] Cheng S Wang H Zhou H. The role of TLR4 on B cell activation and anti-*β* _2_GPI antibody production in the antiphospholipid syndrome. J Immunol Res. (2016) 2016:1719720. doi: 10.1155/2016/1719720, PMID: 27868072 PMC5102736

[160] Wang YH Tsai DY Ko YA Yang TT Lin IY Hung KH. Blimp-1 contributes to the development and function of regulatory B cells. Front Immunol. (2019) 10:1909. doi: 10.3389/fimmu.2019.01909, PMID: 31474988 PMC6702260

[161] Zou F Qiu Y Huang Y Zou H Cheng X Niu Q. Effects of short-chain fatty acids in inhibiting HDAC and activating p38 MAPK are critical for promoting B10 cell generation and function. Cell Death Dis. (2021) 12:582. doi: 10.1038/s41419-021-03880-9, PMID: 34099635 PMC8184914

[162] Fenton TR Gwalter J Ericsson J Gout IT. Histone acetyltransferases interact with and acetylate p70 ribosomal S6 kinases *in vitro* and in *vivo* . Int J Biochem Cell Biol. (2010) 42:359–66. doi: 10.1016/j.biocel.2009.11.022, PMID: 19961954

[163] Fenton TR Gwalter J Cramer R Gout IT. S6K1 is acetylated at lysine 516 in response to growth factor stimulation. Biochem Biophys Res Commun. (2010) 398:400–5. doi: 10.1016/j.bbrc.2010.06.081, PMID: 20599721

[164] Park J Kim M Kang SG Jannasch AH Cooper B Patterson J. Short-chain fatty acids induce both effector and regulatory T cells by suppression of histone deacetylases and regulation of the mTOR–S6K pathway. Mucosal Immunol. (2015) 8:80–93. doi: 10.1038/mi.2014.44, PMID: 24917457 PMC4263689

[165] Liu B-S Cao Y Huizinga TW Hafler DA Toes REM. TLR-mediated STAT3 and ERK activation controls IL-10 secretion by human B cells. Eur J Immunol. (2014) 44:2121–9. doi: 10.1002/eji.201344341, PMID: 24737107

[166] Georgel P Jiang Z Kunz S Janssen E Mols J Hoebe K. Vesicular stomatitis virus glycoprotein G activates a specific antiviral Toll-like receptor 4-dependent pathway. Virology. (2007) 362:304–13. doi: 10.1016/j.virol.2006.12.032, PMID: 17292937

[167] Rassa JC Meyers JL Zhang Y Kudaravalli R Ross SR. Murine retroviruses activate B cells *via* interaction with toll-like receptor 4. Proc Natl Acad Sci U.S.A. (2002) 99:2281–6. doi: 10.1073/pnas.042355399, PMID: 11854525 PMC122356

[168] Lester SN Li K. Toll-like receptors in antiviral innate immunity. J Mol Biol. (2014) 426:1246–64. doi: 10.1016/j.jmb.2013.11.024, PMID: 24316048 PMC3943763

[169] Davie JR. Inhibition of histone deacetylase activity by butyrate. J Nutr. (2003) 133:2485s–93s. doi: 10.1093/jn/133.7.2485S, PMID: 12840228

[170] Belkaid Y Hand TW. Role of the microbiota in immunity and inflammation. Cell. (2014) 157:121–41. doi: 10.1016/j.cell.2014.03.011, PMID: 24679531 PMC4056765

[171] Sun M Wu W Chen L Yang W Huang X Ma C. Microbiota-derived short-chain fatty acids promote Th1 cell IL-10 production to maintain intestinal homeostasis. Nat Commun. (2018) 9:3555. doi: 10.1038/s41467-018-05901-2, PMID: 30177845 PMC6120873

[172] Goritzka M Durant LR Pereira C Salek-Ardakani S Openshaw PJ Johansson C. Alpha/beta interferon receptor signaling amplifies early proinflammatory cytokine production in the lung during respiratory syncytial virus infection. J Virol. (2014) 88:6128–36. doi: 10.1128/jvi.00333-14, PMID: 24648449 PMC4093897

[173] Goritzka M Makris S Kausar F Durant LR Pereira C Kumagai Y. Alveolar macrophage-derived type I interferons orchestrate innate immunity to RSV through recruitment of antiviral monocytes. J Exp Med. (2015) 212:699–714. doi: 10.1084/jem.20140825, PMID: 25897172 PMC4419339

[174] Xu X Lei Y Chen L Zhou H Liu H Jiang J. Phosphorylation of NF-*κ*Bp65 drives inflammation-mediated hepatocellular carcinogenesis and is a novel therapeutic target. J Exp Clin Cancer Res. (2021) 40:253. doi: 10.1186/s13046-021-02062-x, PMID: 34380537 PMC8359590

[175] González-Sanz R Mata M Bermejo-Martín J Álvarez A Cortijo J Melero JA. ISG15 is upregulated in respiratory syncytial virus infection and reduces virus growth through protein ISGylation. J Virol. (2016) 90:3428–38. doi: 10.1128/jvi.02695-15, PMID: 26763998 PMC4794669

[176] Kristiansen H Gad HH Eskildsen-Larsen S Despres P Hartmann R. The oligoadenylate synthetase family: an ancient protein family with multiple antiviral activities. J Interferon Cytokine Res. (2011) 31:41–7. doi: 10.1089/jir.2010.0107, PMID: 21142819

[177] Dhar J Cuevas RA Goswami R Zhu J Sarkar SN Barik S. 2’-5’-Oligoadenylate synthetase-like protein inhibits respiratory syncytial virus replication and is targeted by the viral nonstructural protein 1. J Virol. (2015) 89:10115–9. doi: 10.1128/jvi.01076-15, PMID: 26178980 PMC4577923

[178] Balmer ML Ma EH Bantug GR Grählert J Pfister S Glatter T. Memory CD8^+^ T cells require increased concentrations of acetate induced by stress for optimal function. Immunity. (2016) 44:1312–24. doi: 10.1016/j.immuni.2016.03.016, PMID: 27212436

[179] Buck MD O’Sullivan D Pearce EL. T cell metabolism drives immunity. J Exp Med. (2015) 212:1345–60. doi: 10.1084/jem.20151159, PMID: 26261266 PMC4548052

[180] Halestrap AP Wilson MC. The monocarboxylate transporter family–role and regulation. IUBMB Life. (2012) 64:109–19. doi: 10.1002/iub.572, PMID: 22162139

[181] Watkins PA Maiguel D Jia Z Pevsner J. Evidence for 26 distinct acyl-coenzyme A synthetase genes in the human genome. J Lipid Res. (2007) 48:2736–50. doi: 10.1194/jlr.M700378-JLR200, PMID: 17762044

[182] Sánchez-García FJ Pérez-Hernández CA Rodríguez-Murillo M Moreno-Altamirano MMB. The role of tricarboxylic acid cycle metabolites in viral infections. Front Cell Infect Microbiol. (2021) 11:725043. doi: 10.3389/fcimb.2021.725043, PMID: 34595133 PMC8476952

[183] Scagliola A Mainini F Cardaci S. The tricarboxylic acid cycle at the crossroad between cancer and immunity. Antioxid Redox Signal. (2020) 32:834–52. doi: 10.1089/ars.2019.7974, PMID: 31847530

[184] Zhang J Jia L Lin W Yip YL Lo KW Lau VMY. Epstein-Barr virus-encoded latent membrane protein 1 upregulates glucose transporter 1 transcription *via* the mTORC1/NF-*κ*B signaling pathways. J Virol. (2017) 91:e02168–16. doi: 10.1128/jvi.02168-16, PMID: 28053105 PMC5331802

[185] Philips RL Wang Y Cheon H Kanno Y Gadina M Sartorelli V. The JAK-STAT pathway at 30: Much learned, much more to do. Cell. (2022) 185:3857–76. doi: 10.1016/j.cell.2022.09.023, PMID: 36240739 PMC9815833

[186] Afonina IS Tynan GA Logue SE Cullen SP Bots M Lüthi AU. Granzyme B-dependent proteolysis acts as a switch to enhance the proinflammatory activity of IL-1*α* . Mol Cell. (2011) 44:265–78. doi: 10.1016/j.molcel.2011.07.037, PMID: 22017873 PMC3319689

[187] Roediger WE. Utilization of nutrients by isolated epithelial cells of the rat colon. Gastroenterology. (1982) 83:424–9. doi: 10.1016/S0016-5085(82)80339-9, PMID: 7084619

[188] Wu W Sun M Chen F Cao AT Liu H Zhao Y. Microbiota metabolite short-chain fatty acid acetate promotes intestinal IgA response to microbiota which is mediated by GPR43. Mucosal Immunol. (2017) 10:946–56. doi: 10.1038/mi.2016.114, PMID: 27966553 PMC5471141

[189] Singh N Thangaraju M Prasad PD Martin PM Lambert NA Boettger T. Blockade of dendritic cell development by bacterial fermentation products butyrate and propionate through a transporter (Slc5a8)-dependent inhibition of histone deacetylases. J Biol Chem. (2010) 285:27601–8. doi: 10.1074/jbc.M110.102947, PMID: 20601425 PMC2934627

[190] Christ A Lauterbach M Latz E. Western diet and the immune system: an inflammatory connection. Immunity. (2019) 51:794–811. doi: 10.1016/j.immuni.2019.09.020, PMID: 31747581

[191] Nelson BN Friedman JE. Developmental programming of the fetal immune system by maternal western-style diet: mechanisms and implications for disease pathways in the offspring. Int J Mol Sci. (2024) 25:5951. doi: 10.3390/ijms25115951, PMID: 38892139 PMC11172957

[192] Belkaid Y Harrison OJ. Homeostatic immunity and the microbiota. Immunity. (2017) 46:562–76. doi: 10.1016/j.immuni.2017.04.008, PMID: 28423337 PMC5604871

[193] Ansaldo E Farley TK Belkaid Y. Control of immunity by the microbiota. Annu Rev Immunol. (2021) 39:449–79. doi: 10.1146/annurev-immunol-093019-112348, PMID: 33902310

[194] Macfarlane GT Gibson GR Cummings JH. Comparison of fermentation reactions in different regions of the human colon. J Appl Bacteriol. (1992) 72:57–64. doi: 10.1111/j.1365-2672.1992.tb04882.x, PMID: 1541601

[195] Mukhopadhya I Louis P. Gut microbiota-derived short-chain fatty acids and their role in human health and disease. Nat Rev Microbiol. (2025). doi: 10.1038/s41579-025-01183-w, PMID: 40360779

[196] van der Beek CM Bloemen JG van den Broek MA Lenaerts K Venema K Buurman WA. Hepatic uptake of rectally administered butyrate prevents an increase in systemic butyrate concentrations in humans. J Nutr. (2015) 145:2019–24. doi: 10.3945/jn.115.211193, PMID: 26156796

[197] van der Beek Christina M Canfora Emanuel E Lenaerts K Troost Freddy J Olde Damink SWM Holst Jens J. Distal, not proximal, colonic acetate infusions promote fat oxidation and improve metabolic markers in overweight/obese men. Clin Sci (Lond). (2016) 130:2073–82. doi: 10.1042/cs20160263, PMID: 27439969

[198] Donovan JD Bauer L Fahey GC Lee Y. *In vitro* digestion and fermentation of microencapsulated tributyrin for the delivery of butyrate. J Food Sci. (2017) 82:1491–9. doi: 10.1111/1750-3841.13725, PMID: 28485486

[199] Annison G Illman RJ Topping DL. Acetylated, propionylated or butyrylated starches raise large bowel short-chain fatty acids preferentially when fed to rats. J Nutr. (2003) 133:3523–8. doi: 10.1093/jn/133.11.3523, PMID: 14608068

[200] Mariño E Richards JL McLeod KH Stanley D Yap YA Knight J. Gut microbial metabolites limit the frequency of autoimmune T cells and protect against type 1 diabetes. Nat Immunol. (2017) 18:552–62. doi: 10.1038/ni.3713, PMID: 28346408

[201] Hutkins R Walter J Gibson GR Bedu-Ferrari C Scott K Tancredi DJ. Classifying compounds as prebiotics — scientific perspectives and recommendations. Nat Rev Gastroenterol Hepatol. (2025) 22:54–70. doi: 10.1038/s41575-024-00981-6, PMID: 39358591

[202] Wolever TM ter Wal P Spadafora P Robb P. Guar, but not psyllium, increases breath methane and serum acetate concentrations in human subjects. Am J Clin Nutr. (1992) 55:719–22. doi: 10.1093/ajcn/55.3.719, PMID: 1312763

[203] Rahat-Rozenbloom S Fernandes J Cheng J Gloor GB Wolever TM. The acute effects of inulin and resistant starch on postprandial serum short-chain fatty acids and second-meal glycemic response in lean and overweight humans. Eur J Clin Nutr. (2017) 71:227–33. doi: 10.1038/ejcn.2016.248, PMID: 27966565 PMC5298923

[204] Vinelli V Biscotti P Martini D Del Bo’ C Marino M Meroño T. Effects of dietary fibers on short-chain fatty acids and gut microbiota composition in healthy adults: a systematic review. Nutrients. (2022) 14:2559. doi: 10.3390/nu14132559, PMID: 35807739 PMC9268559

[205] Markowiak-Kopeć P Śliżewska K. The effect of probiotics on the production of short-chain fatty acids by human intestinal microbiome. Nutrients. (2020) 12:1107. doi: 10.3390/nu12041107, PMID: 32316181 PMC7230973

[206] El-Salhy M Valeur J Hausken T Gunnar Hatlebakk J. Changes in fecal short-chain fatty acids following fecal microbiota transplantation in patients with irritable bowel syndrome. Neurogastroenterol Motil. (2021) 33:e13983. doi: 10.1111/nmo.13983, PMID: 32945066 PMC7900992

[207] Smits LP Bouter KE de Vos WM Borody TJ Nieuwdorp M. Therapeutic potential of fecal microbiota transplantation. Gastroenterology. (2013) 145:946–53. doi: 10.1053/j.gastro.2013.08.058, PMID: 24018052

[208] Arnold J Glazier J Mimee M. Genetic engineering of resident bacteria in the gut microbiome. J Bacteriol. (2023) 205:e00127–23. doi: 10.1128/jb.00127-23, PMID: 37382533 PMC10367592

[209] Kim KN Yao Y Ju SY. Short chain fatty acids and fecal microbiota abundance in humans with obesity: a systematic review and Meta-analysis. Nutrients. (2019) 11:2512. doi: 10.3390/nu11102512, PMID: 31635264 PMC6835694

[210] Wen L Ley RE Volchkov PY Stranges PB Avanesyan L Stonebraker AC. Innate immunity and intestinal microbiota in the development of Type 1 diabetes. Nature. (2008) 455:1109–13. doi: 10.1038/nature07336, PMID: 18806780 PMC2574766

[211] Miyamoto J Kasubuchi M Nakajima A Irie J Itoh H Kimura I. The role of short-chain fatty acid on blood pressure regulation. Curr Opin Nephrol Hypertens. (2016) 25:379–83. doi: 10.1097/mnh.0000000000000246, PMID: 27490782

[212] McNabney SM Henagan TM. Short chain fatty acids in the colon and peripheral tissues: a focus on butyrate, colon cancer, obesity and insulin resistance. Nutrients. (2017) 9:1348. doi: 10.3390/nu9121348, PMID: 29231905 PMC5748798

[213] Rossini V Tolosa-Enguis V Frances-Cuesta C Sanz Y. Gut microbiome and anti-viral immunity in COVID-19. Crit Rev Food Sci Nutr. (2024) 64:4587–602. doi: 10.1080/10408398.2022.2143476, PMID: 36382631

[214] Wang Q Fang Z Li L Wang H Zhu J Zhang P. *Lactobacillus mucosae* exerted different antiviral effects on respiratory syncytial virus infection in mice. Front Microbiol. (2022) 13:1001313. doi: 10.3389/fmicb.2022.1001313, PMID: 36090099 PMC9459143

[215] Cheong YJ Trefely S. Divergent roles for propionate and butyrate in colorectal cancer epigenetics. Nat Metab. (2025) 7:11–3. doi: 10.1038/s42255-024-01186-6, PMID: 39789353

[216] Gilbert SF Bosch TC Ledón-Rettig C. Eco-Evo-Devo: developmental symbiosis and developmental plasticity as evolutionary agents. Nat Rev Genet. (2015) 16:611–22. doi: 10.1038/nrg3982, PMID: 26370902

[217] Shapira M. Gut microbiotas and host evolution: scaling up symbiosis. Trends Ecol Evol. (2016) 31:539–49. doi: 10.1016/j.tree.2016.03.006, PMID: 27039196

[218] Sikder MAA Rashid RB Ahmed T Sebina I Howard DR Ullah MA. Maternal diet modulates the infant microbiome and intestinal Flt3L necessary for dendritic cell development and immunity to respiratory infection. Immunity. (2023) 56:1098–114.e10. doi: 10.1016/j.immuni.2023.03.002, PMID: 37003256

[219] Brown AJ Jupe S Briscoe CP. A family of fatty acid binding receptors. DNA Cell Biol. (2005) 24:54–61. doi: 10.1089/dna.2005.24.54, PMID: 15684720

[220] Stoddart LA Smith NJ Milligan G International Union of Pharmacology. LXXI. Free fatty acid receptors FFA1, -2, and -3: pharmacology and pathophysiological functions. Pharmacol Rev. (2008) 60:405–17. doi: 10.1124/pr.108.00802, PMID: 19047536

[221] Tolhurst G Heffron H Lam YS Parker HE Habib AM Diakogiannaki E. Short-chain fatty acids stimulate glucagon-like peptide-1 secretion *via* the G-protein-coupled receptor FFAR2. Diabetes. (2012) 61:364–71. doi: 10.2337/db11-1019, PMID: 22190648 PMC3266401

[222] Kobayashi M Mikami D Kimura H Kamiyama K Morikawa Y Yokoi S. Short-chain fatty acids, GPR41 and GPR43 ligands, inhibit TNF-*α*-induced MCP-1 expression by modulating p38 and JNK signaling pathways in human renal cortical epithelial cells. Biochem Biophys Res Commun. (2017) 486:499–505. doi: 10.1016/j.bbrc.2017.03.071, PMID: 28322790

[223] Park J Wang Q Wu Q Mao-Draayer Y Kim CH. Bidirectional regulatory potentials of short-chain fatty acids and their G-protein-coupled receptors in autoimmune neuroinflammation. Sci Rep. (2019) 9:8837. doi: 10.1038/s41598-019-45311-y, PMID: 31222050 PMC6586800

[224] Kimura I Miyamoto J Ohue-Kitano R Watanabe K Yamada T Onuki M. Maternal gut microbiota in pregnancy influences offspring metabolic phenotype in mice. Science. (2020) 367:eaaw8429. doi: 10.1126/science.aaw8429, PMID: 32108090

[225] Liu JL Segovia I Yuan XL Gao ZH. Controversial roles of gut microbiota-derived short-chain fatty acids (SCFAs) on pancreatic *β*-cell growth and insulin secretion. Int J Mol Sci. (2020) 21:910. doi: 10.3390/ijms21030910, PMID: 32019155 PMC7037182

[226] Ahmed K Tunaru S Offermanns S. GPR109A, GPR109B and GPR81, a family of hydroxy-carboxylic acid receptors. Trends Pharmacol Sci. (2009) 30:557–62. doi: 10.1016/j.tips.2009.09.001, PMID: 19837462

[227] Fujino T Kondo J Ishikawa M Morikawa K Yamamoto TT. Acetyl-CoA synthetase 2, a mitochondrial matrix enzyme involved in the oxidation of acetate. J Biol Chem. (2001) 276:11420–6. doi: 10.1074/jbc.M008782200, PMID: 11150295

[228] Comerford SA Huang Z Du X Wang Y Cai L Witkiewicz AK. Acetate dependence of tumors. Cell. (2014) 159:1591–602. doi: 10.1016/j.cell.2014.11.020, PMID: 25525877 PMC4272450

[229] Narita T Weinert BT Choudhary C. Functions and mechanisms of non-histone protein acetylation. Nat Rev Mol Cell Biol. (2019) 20:156–74. doi: 10.1038/s41580-018-0081-3, PMID: 30467427

[230] You D Wang MM Yin BC Ye BC. Precursor supply for erythromycin biosynthesis: engineering of propionate assimilation pathway based on propionylation modification. ACS Synth Biol. (2019) 8:371–80. doi: 10.1021/acssynbio.8b00396, PMID: 30657660

[231] Tomasova L Grman M Ondrias K Ufnal M. The impact of gut microbiota metabolites on cellular bioenergetics and cardiometabolic health. Nutr Metab. (2021) 18:72. doi: 10.1186/s12986-021-00598-5, PMID: 34266472 PMC8281717

[232] Hao F Tian M Zhang X Jin X Jiang Y Sun X. Butyrate enhances CPT1A activity to promote fatty acid oxidation and iTreg differentiation. Proc Natl Acad Sci USA. (2021) 118:e2014681118. doi: 10.1073/pnas.2014681118, PMID: 34035164 PMC8179238

[233] Chowdhury NP Kahnt J Buckel W. Reduction of ferredoxin or oxygen by flavin-based electron bifurcation in *Megasphaera elsdenii* . FEBS J. (2015) 282:3149–60. doi: 10.1111/febs.13308, PMID: 25903584

[234] Wang L Zong Z Liu Y Zheng M Li D Wang C. Metabolic engineering of *Yarrowia lipolytica* for the biosynthesis of crotonic acid. Bioresour Technol. (2019) 287:121484. doi: 10.1016/j.biortech.2019.121484, PMID: 31121443

[235] Trefely S Lovell CD Snyder NW Wellen KE. Compartmentalised acyl-CoA metabolism and roles in chromatin regulation. Mol Metab. (2020) 38:100941. doi: 10.1016/j.molmet.2020.01.005, PMID: 32199817 PMC7300382

[236] Xie L Xue F Cheng C Sui W Zhang J Meng L. Cardiomyocyte-specific knockout of ADAM17 alleviates doxorubicin-induced cardiomyopathy *via* inhibiting TNF*α*-TRAF3-TAK1-MAPK axis. Sig Transduct Target Ther. (2024) 9:273. doi: 10.1038/s41392-024-01977-z, PMID: 39406701 PMC11480360

